# Similarities and differences between multivariate patterns of cognitive and socio-cognitive deficits in schizophrenia, bipolar disorder and related risk

**DOI:** 10.1038/s41537-023-00337-0

**Published:** 2023-02-17

**Authors:** Alessandra Raio, Giulio Pergola, Antonio Rampino, Marianna Russo, Enrico D’Ambrosio, Pierluigi Selvaggi, Valerie De Chiara, Mario Altamura, Flora Brudaglio, Alessandro Saponaro, Domenico Semisa, Alessandro Bertolino, Linda A. Antonucci, Giuseppe Blasi, the Apulian Network on Risk for Psychosis, Anna Manzari, Angela Carofiglio, Giuseppe Barrasso, Antonello Bellomo, Ivana Leccisotti, Melania Di Fino, Ileana Andriola, Teresa Claudia Pennacchio

**Affiliations:** 1 Department of Translational Biomedicine and Neuroscience - University of Bari Aldo Moro, Bari, Italy; 2 Psychiatry Unit - University Hospital, Bari, Italy; 3 Department of Psychosis Studies, Institute of Psychiatry, Psychology and Neuroscience, King’s College London, London, SE5 8AF UK; 4 Department of Clinical and Experimental Medicine, Psychiatry Unit, University of Foggia, Foggia, Italy; 5 Department of Mental Health, ASL BAT, Andria, Italy; 6 Department of Mental Health, ASL BR, Brindisi, Italy; 7 Department of Mental Health, ASL BA, Bari, Italy

**Keywords:** Psychosis, Human behaviour

## Abstract

Cognition and social cognition anomalies in patients with bipolar disorder (BD) and schizophrenia (SCZ) have been largely documented, but the degree of overlap between the two disorders remains unclear in this regard. We used machine learning to generate and combine two classifiers based on cognitive and socio-cognitive variables, thus delivering unimodal and multimodal signatures aimed at discriminating BD and SCZ from two independent groups of Healthy Controls (HC1 and HC2 respectively). Multimodal signatures discriminated well between patients and controls in both the HC1-BD and HC2-SCZ cohorts. Although specific disease-related deficits were characterized, the HC1 vs. BD signature successfully discriminated HC2 from SCZ, and vice-versa. Such combined signatures allowed to identify also individuals at First Episode of Psychosis (FEP), but not subjects at Clinical High Risk (CHR), which were classified neither as patients nor as HC. These findings suggest that both trans-diagnostic and disease-specific cognitive and socio-cognitive deficits characterize SCZ and BD. Anomalous patterns in these domains are also relevant to early stages of disease and offer novel insights for personalized rehabilitative programs.

## Introduction

Schizophrenia and bipolar disorder are two severe brain diseases that heavily compromise quality of life and personal functioning^1,2^. Despite their traditional nosological discrimination^3^, cross-domain evidence suggests a quite large degree of clinical overlap. Indeed, patients often do not fit completely within the boundaries of a single disorder and show a mixture of psychopathological features traditionally associated with both these illnesses^4,5^. Therefore, rather than considering schizophrenia and bipolar disorder as two separate diagnostic entities, recent views have hypothesized that they lie on a psychopathological continuum^6^. This view is further supported by growing evidence highlighting partially shared genetic risk between the two diseases^7,8^. Thus, partially-shared phenotypes^9^ may characterize these brain disorders, under the influence of partially-shared genetic risk factors.

Among phenotypes in common between patients with schizophrenia (SCZ) and with bipolar disorder (BD), those related to cognitive and socio-cognitive impairments^10,11^ play a key role. Indeed, such correlates are crucially associated with both the diseases^12^. Univariate literature^13,14^ is relatively consistent in showing more quantitative (i.e., in terms of severity of impairments) than qualitative (i.e., in terms of differentially impaired domains) cognitive differences between bipolar disorder and schizophrenia. Typically, SCZ exhibit more severe and pervasive core cognitive deficits, which affect multiple domains including processing speed^15^, verbal fluency^16^, episodic and working memory^17^, and cognitive flexibility^18^. These alterations usually emerge in the context of a global intellectual impairment^10,19^ and are tightly associated with negative symptoms and disorganization, crucially contributing to the typical increased functional disability in SCZ^20^.

Also BD experience specific alterations affecting different cognitive domains^13,14,21^, but to a lesser extent and within a framework of relatively preserved general intelligence, likely reflecting better premorbid functioning^2^. This pattern of milder cognitive impairment in bipolar disorder emerges across a broad range of domains, including attention, verbal memory, working memory, and executive functioning^10,22^. This body of evidence suggests that BD and SCZ could be differentiated at the cognitive level only quantitatively, and not qualitatively. Interestingly, this seems to be the case also for social cognition. In this regard, meta-analytic findings reveal a stable pattern of socio-cognitive deficits across domains, including recognition and perception of basic socio-emotional cues, social inference^23^ as well as theory of mind^11^, with SCZ showing the same socio-cognitive abnormalities of BD, but to a greater extent^11,23^. Taken together, this literature suggests that SCZ and BD are difficult to distinguish based on cognitive and socio-cognitive characteristics. Indeed, these impairments define a dimensional phenotype occurring among psychosis spectrum disorders^24^. Furthermore, subclinical signs identifying risk conditions for psychosis spectrum disorders are paralleled by both cognitive and socio-cognitive deficits^22^. In this regard, previous studies have highlighted that individuals at clinical high risk for psychosis (i.e., at-risk mental state for psychosis—ARMS)^25^ and bipolar disorder (i.e., bipolar at-risk—BAR)^26^ show a large extent of quantitative and qualitative similarities in the occurrence of early cognitive^27^ and socio-cognitive impairments^28^.

However, all this literature is based on univariate statistical approaches. Compared with these methods, which may leverage knowledge only at the group-level^29^, techniques based on multivariate approaches, like Machine learning (ML), use information from multiple data sources to classify groups of subjects^30^ based on parsimonious sets of variables (i.e., signatures). This approach allows classification of individuals at the single-subject level, avoiding a group vs. group perspective^31,32^. Thus, it may contribute to successfully identify individualized and fine-grained patterns of cognitive and socio-cognitive anomalies in SCZ and BD, as well as to investigate their diagnosis-related or trans-diagnostic relevance.

**Fig. 1:**
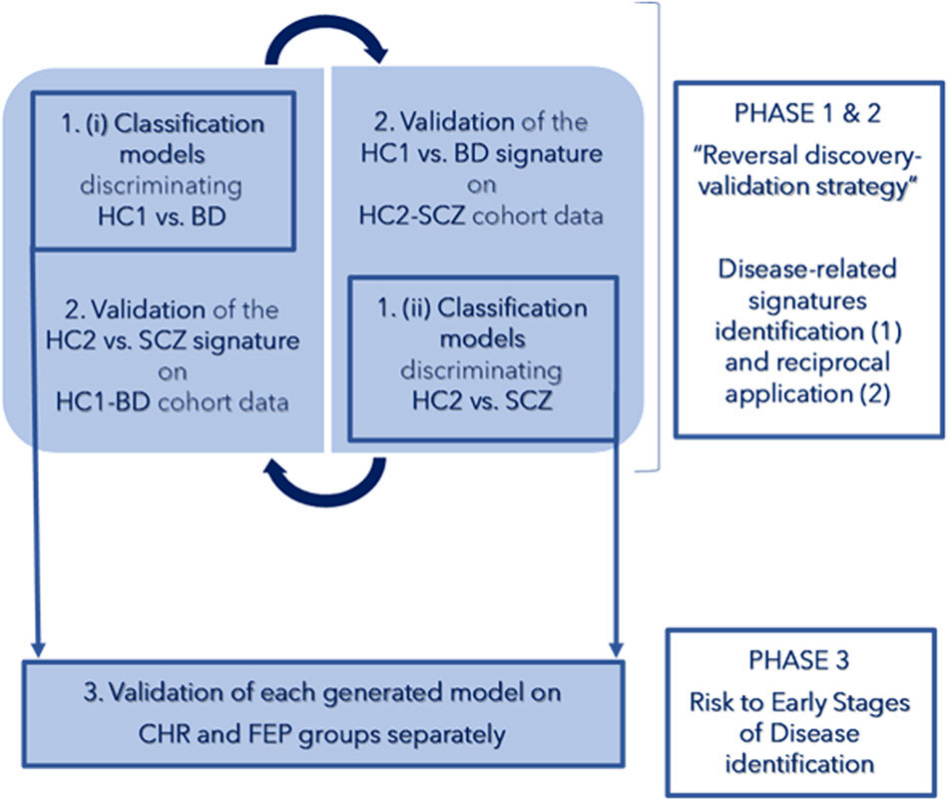
Outline of the study design. To detect cognitive and socio-cognitive similarities and differences between schizophrenia and bipolar disorder, we employed a “reversal discovery-validation strategy”, consisting of two phases: (i) in Phase 1, we generated unimodal and multimodal signatures based on cognitive and socio-cognitive features aimed at discriminating two independent groups of Healthy Controls from two groups of patients suffering from Bipolar Disorder and Schizophrenia, respectively; (ii) in Phase 2, we applied the disease-related models generated in Phase 1 in each cohort to the other one data. In Phase 3, to test the generalizability of disease-related signatures on populations at risk or at early stages of psychosis, we applied the discovery models generated in Phase 1 also to cognitive and socio-cognitive data collected on Clinical High-Risk and First Episode of Psychosis individuals. BD = patients with Bipolar Disorder; CHR = participants with Clinical High Risk for psychosis; FEP = participants at First Episode of Psychosis; HC1 = Healthy Controls (group 1); HC2 = Healthy Controls (group 2); SCZ = patients with Schizophrenia.

This study aimed to identify at the single-subject level data-driven cognitive and socio-cognitive signatures of BD and SCZ, and to investigate their trans-diagnostic or diagnosis-specific relevance. Another aim was to investigate if such signatures are also relevant to risk conditions for these diseases or their early stages. With this purpose, we devised a “reversal discovery-validation” ML strategy (Fig. 1—Phase 1 & 2). First, we generated three ML algorithms, hereby called “classifiers”. The first classifier was based on cognition (unimodal classifier), the second on social cognition (unimodal classifier), and the third was a higher-order classifier that learned from the combined decisions of the cognitive and socio-cognitive classifiers (multimodal classifier). Then, we tested the classifiers’ accuracy in predicting the membership of each individual (i) within a cohort including a group of healthy controls (Healthy Controls - group 1: HC1) and BD as well as (ii) within an independent cohort including another group of healthy controls (Healthy Controls - group 2: HC2) and SCZ (Fig. 1—Phase 1; Methods, “Phase 1: generation of unimodal and multimodal classifiers”). Thus, to test the degree of overlaps and specificities of BD- and SCZ-related models, we applied the uni- and multimodal classifiers predicting HC1 and BD memberships to the HC2-SCZ cohort, and vice-versa (Fig. 1—Phase 2; Methods, “Phase 2: reciprocal validation of the generated unimodal and multimodal classifiers”). Finally, to investigate the generalization of our unimodal and multimodal classifiers to earlier stages of disease and psychosis risk, models were applied to independent cohorts of individuals at clinical risk for psychosis (Clinical High Risk—CHR) or earlier stages of disease (First Episode of Psychosis—FEP) via Out Of sample Cross-Validation (OOCV) (Fig. 1—Phase 3; Methods, “Phase 3: investigating the generalization of unimodal and multimodal classifiers”).

## Results

### Demographic and clinical differences between healthy controls and patients

T-test and χ^2^ revealed that HC1 differed from BD in terms of age, socio-economic status, current and premorbid IQ (all *p* < 0.001) (Table 1A). On the other hand, HC2 differed from SCZ in terms of gender, age, socio-economic status, current and premorbid IQ (all *p* < 0.001) (Table 1B). Furthermore, ANOVAs indicated that CHR and FEP differed from patients and control groups of both HC1-BD and HC2-SCZ cohorts in terms of age, socio-economic status, current and premorbid IQ (all *p* < 0.05) (Table 2).

**Table 1:** Demographic and clinical characteristics of: (**A**) Healthy Controls (group 1) compared with Bipolar Disorder patients; (**B**) Healthy Controls (group 2) compared with Schizophrenia patients.

A. HC1-BD cohort	HC1 + BD (mean ± SD)	HC1 (mean ± SD)	BD (mean ± SD)	HC1 vs. BD (T/χ² [*p*-value])
Sample size	154	95	59	n.a.
Gender ratio (M/F)	66/88	36/59	30/29	1.99 [0.16]
Age	30.9 ± 11.7	26.57 ± 7.55	38.08 ± 13.66	−5.9 [<0.001*]
Socio-Economic Status	37.4 ± 17.4	41.25 ± 16.57	31.19 ± 17.06	3.6 [<0.001*]
Current IQ	102.2 ± 15.8	110.13 ± 11.54	89.32 ± 13.25	10.3 [<0.001*]
Premorbid IQ	113.5 ± 5.9	116 ± 2.70	109.47 ± 7.29	6.6 [<0.001*]
GAF total score	n.a.	n.a.	64.3 ± 6.2	n.a.
Lithium carbonate Equivalent dose	n.a.	n.a.	0.79 ± 0.39	n.a.
PANSS total score	n.a.	n.a.	48.6 ± 6.4	n.a.
YMRS total score	n.a.	n.a.	3.9 ± 1.3	n.a.
B. HC2-SCZ cohort	HC2 + SCZ (mean ± SD)	HC2 (mean ± SD)	SCZ (mean ± SD)	HC2 vs. SCZ (T/χ² [*p*-value])
Sample size	313	195	118	n.a.
Gender ratio (M/F)	117/136	88/107	89/29	26.24 [<0.001*]
Age	28.35 ± 8.12	26.40 ± 6.87	31.58 ± 8.99	−5.4 [<0.001*]
Socio-Economic Status	36.12 ± 17.54	39.37 ± 16.86	30.75 ± 17.40	4.3 [<0.001*]
Current IQ	96.96 ± 18.67	108.12 ± 10.57	78.63 ± 14.03	21.1 [<0.001*]
Premorbid IQ	112.36 ± 6.66	115.33 ± 3.36	107.46 ± 7.78	10.4 [<0.001*]
GAF total score	n.a.	n.a.	56.10 ± 8.89	n.a.
Chlorpromazine equivalent dose	n.a.	n.a.	118.76 ± 34.19	n.a.
PANSS total score	n.a.	n.a.	99.08 ± 26.55	n.a.

Significant between-groups differences (*p* < 0.05) are marked with (*).

*BD* patients with Bipolar Disorder, *HC1* Healthy Controls (group 1), *HC2* Healthy Controls (group 2), *IQ* Intelligence Quotient, *M/F* males/females, *n.a.* not assessed, *PANSS* Positive and Negative Symptoms Scale, *SCZ* patients with Schizophrenia, *SD* standard deviation, *YMRS* Young Mania Rating Scale.

**Table 2:** Demographic and clinical characteristics of: [**A**] Clinical High-Risk individuals; [**B**] First Episode of Psychosis individuals.

A	Clinical High-Risk	ANOVA comparison between CHR-HC1-BD (F [*p*])	ANOVA comparison between CHR-HC2-SCZ (F [*p*])
Sample size	35	n.a.	n.a.
Gender ratio [M/F]	24/11	n.a.	n.a.
Age	19.80 ± 4.5	46.2 [<0.001*]	283.7 [<0.001*]
Socio-Economic Status	31.6 ± 16.6	8.2 [<0.001*]	10.5 [<0.001*]
Current IQ	87.4 ± 11.6	74.9 [<0.001*]	223.3 [<0.001*]
Premorbid IQ	107.4 ± 5.9	48.9 [<0.001*]	87.7 [<0.001*]
B	First Episode of Psychosis	ANOVA comparison between FEP-HC1-BD (F [*p*])	ANOVA comparison between FEP-HC2-SCZ (F [*p*])
Sample size	29	n.a.	n.a.
Gender ratio [M/F]	17/12	n.a.	n.a.
Age	22.4 ± 5.2	35.1 [<0.001*]	275.1 [<0.001*]
Socio-Economic Status	35.9 ± 17.1	6.6 [0.002*]	9.3 [<0.001*]
Current IQ	73.1 ± 15.5	111.2 [<0.001*]	259.9 [<0.001*]
Premorbid IQ	106.8 ± 6.1	49.4 [<0.001*]	88.2 [<0.001*]

ANOVA analyses were performed to compare individuals at early stages of disease with the groups included in the Healthy Controls (group 1) – Bipolar Disorder patients cohort and in the Healthy Controls (group 2) – Schizophrenia patients cohort. Significant between-groups differences (*p* < 0.05) are marked with [*].

*BD* patients with Bipolar Disorder, C*HR* individuals at Clinical High-Risk, *FEP* patients at First Episode of Psychosis, *HC1* Healthy Controls (group 1), *HC2* Healthy Controls (group 2), *IQ* Intelligence Quotient, *M/F* males/females, *n.a*. not assessed, *SCZ* patients with Schizophrenia, *SD* standard deviation.

### Machine learning

#### Phase 1: classification models between Healthy Controls (group 1) and Bipolar Disorder patients

**Fig. 2:**
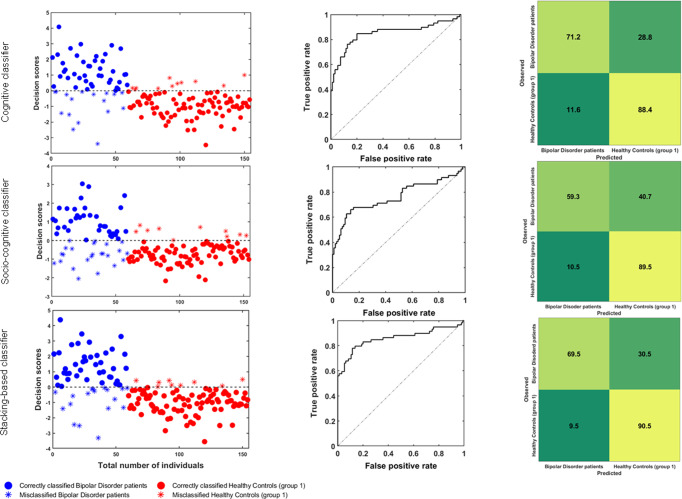
Performance metrics (classification plots, Receiver Operating Characteristic curves, and confusion matrices) of the classifiers discriminating between Healthy Controls (group 1) and Bipolar Disorder patients. First row: cognitive classifier; second row: socio-cognitive classifier; third row: stacking-based classifier.

In order to classify HC1 vs. BD, we built three separate within-cohort Support Vector Machine (SVM) algorithms. SVM is a margin-based statistical technique of supervised learning able to discriminate individuals into two (or more) groups by establishing a linear space of classification (i.e., a hyperplane) on the basis of specific cases called support vectors^29^. The first algorithm was based on performance at cognitive tests, whereas the second incorporated socio-cognitive measures. The third was a multimodal SVM, i.e., stacking-based, which allows to combine the unimodal classifiers^33^, integrating their multiple learned decision values and then learning again from this meta-information (Methods, “Phase 1: generation of unimodal and multimodal classifiers”). All the generated classifiers correctly discriminated BD from HC1 better than chance with a permuted significance of *p* < 0.05. In particular, we used balanced accuracy (BAC) as a measure of model performance, which refers to accuracy calculated in terms of true positive and negative cases, balanced by the sample size of each positive and negative group. BAC is used to optimize performance in models with unbalanced sample sizes^29^. According to this measure, the stacking-based model had the best classification performance, assigning membership of each individual to BD and HC1 with 80.0% BAC (*p* = 0.02). The cognitive classifier revealed a cross-validated BAC of 79.8% (*p* = <0.001), while BAC of the socio-cognitive classifier was 74.4% (*p* = <0.001) (for detailed classification results, see Table 3A and Fig. 2). To investigate putative association of predicted membership with psychopharmacological treatment or symptom scores, we extracted decision scores from the best-performing classifier (i.e., the stacking-based). Decision scores represent the individual geometric distance from the decision boundary. This resulted in a decision value and a predicted classification label per participant^32^. Spearman’s test revealed no significant associations between decision scores extracted from the stacking-based classifier and lithium carbonate equivalent dose (Spearman’s rho = −0.103, *p* = 0.44) or mania as assessed by Young Mania Rating Scale (YMRS)^34^ (Spearman’s rho = 0.062, *p* = 0.641) for BD.

**Table 3:** Reversal discovery-validation strategy classification performance.

	True negatives	True positives	False negatives	False positives	Sensitivity	Specificity	Balanced accuracy (%)	Area under the curve	Positive predictive value	Negative predictive value	Positive likelihood ratio
Classification: Healthy Controls (group 1) vs. Bipolar Disorder patients											
Cognitive classifier	84	42	17	11	71.2	88.4	79.8	0.85	79.2	83.2	6.1
Socio-cognitive classifier	85	35	24	10	59.3	89.5	74.4	0.76	77.8	78.0	5.6
Stacking-based multimodal classifier	86	41	18	9	69.5	90.5	80	0.86	82	82.7	7.3
Classification: Healthy Controls (group 2) vs. Schizophrenia patients											
Cognitive classifier	175	91	27	20	77.1	87.9	83.4	0.90	82	86.6	7.5
Socio-cognitive classifier	171	82	36	24	69.5	87.7	78.6	0.85	77.4	82.6	5.6
Stacking-based multimodal classifier	179	92	26	16	78	91.8	84.9	0.92	85.2	87.3	9.5

A: Validated classification performance of unimodal and stacking-based multimodal classifiers in the cohort including Healthy Controls (group 1) and Bipolar Disorder patients. B: Validated classification performance of unimodal and stacking-based multimodal classifiers in the cohort including Healthy Controls (group 2) and Schizophrenia patients.

**Fig. 3:**
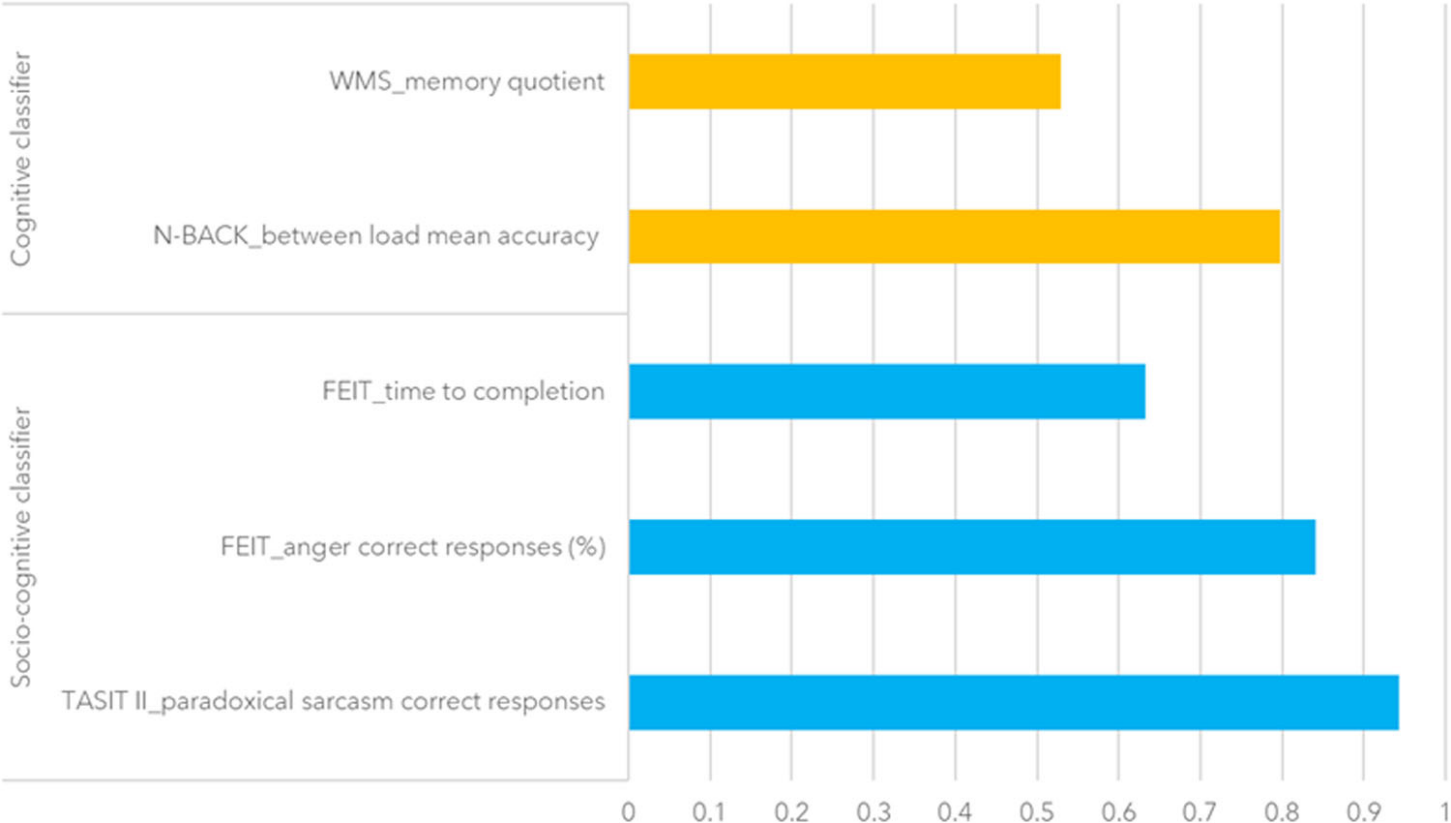
Probability of each feature for being selected in the Machine Learning Cross-Validation framework for the cognitive and the socio-cognitive classifiers in the cohort including Healthy Controls (group 1) and Bipolar Disorder patients. Scores closer to 1 represent a higher probability of being selected for decisions by the Support Vector Machine algorithm. Only features with a selection probability >0.5 are shown (complete selection probability results for the whole pool of cognitive and socio-cognitive features are available in SF1A and 1B, respectively). FEIT = Facial Emotion Identification Test; N = number; TASIT II = The Awareness of Social Inference Test – Section II; WMS = Wechsler Memory Scale.

Then, we looked at the features with the highest probability of being selected for HC1 vs. BD classification. With regard to the cognitive classifier, they were the mean accuracy (as the correct response percentage) between loads at the N-back working memory task^35^, and the global memory IQ score^36^ (Fig. 3). With regard to the socio-cognitive classifier, they were the total score for the ability to infer paradoxical sarcasm during videotaped interactions, assessed by The Awareness of Social Inference Test (TASIT)^37^, the accuracy in identifying anger facial expressions, assessed by the Facial Emotion Identification Test (FEIT)^38^, and the total reaction time at the FEIT (Fig. 3).

#### Phase 1: classification models between Healthy Controls (group 2) and Schizophrenia patients

**Fig. 4:**
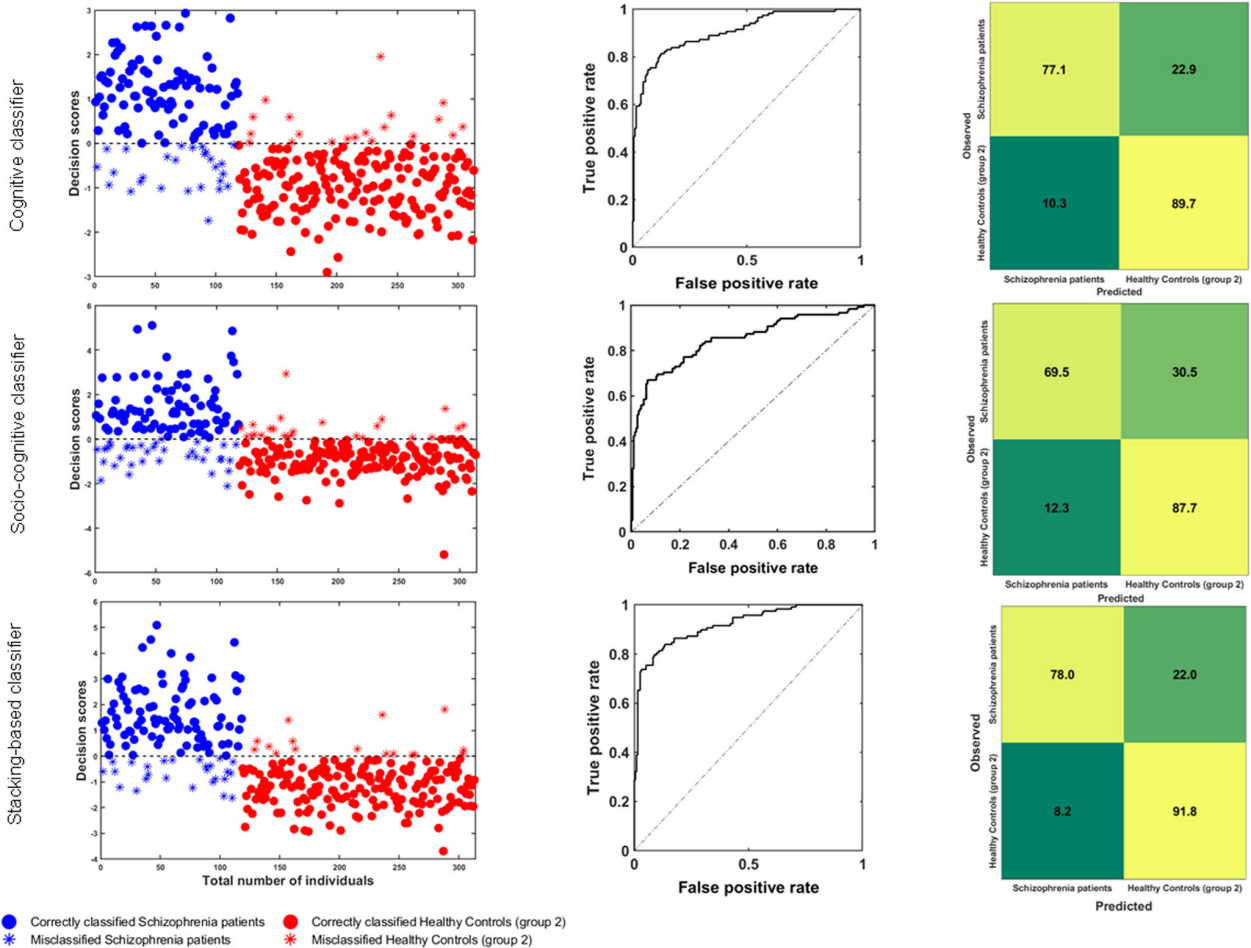
Performance metrics (classification plots, Receiver Operating Characteristic curves, and confusion matrices) of the classifiers discriminating between Healthy Controls (group 2) and Schizophrenia patients. First row: cognitive classifier; second row: socio-cognitive classifier; third row: stacking-based classifier.

We used the same method described for HC1 vs. BD discrimination to build two unimodal and one stacking-based SVM algorithms for HC2 vs. SCZ classification. Here, all the generated models correctly classified each individual as SCZ or HC2 better than chance with a permuted significance <0.001. As in the HC1 vs. BD analysis, the stacking-based model performed better than the two unimodal classifiers with 84.9% BAC (*p* = <0.001), while cross-validated BAC was 83.4% (*p* = <0.001) for the cognitive classifier, and 78.6% (*p* = <0.001) for the socio-cognitive classifier (for detailed classification results, see Table 3B and Fig. 4). Spearman’s test revealed no significant associations between decision scores extracted from the best-performing classifier (i.e., the stacking-based) and chlorpromazine equivalents (Spearman’s rho = 0.019, *p* = 0.83) or psychosis-related psychopathology as assessed by Positive And Negative Symptoms Scale (PANSS)^39^ total score (Spearman’s rho = 0.020, *p* = 0.828) for SCZ.

**Fig. 5:**
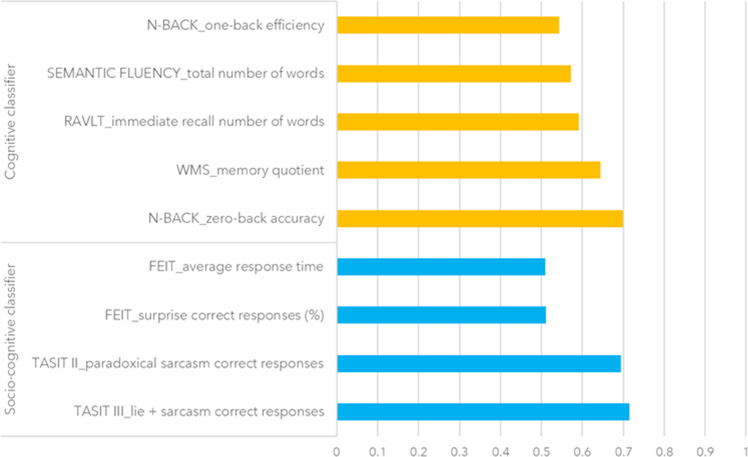
Probability of each feature for being selected in the Machine Learning Cross-Validation framework for the cognitive and the socio-cognitive classifiers in the cohort including Healthy Controls (group 2) and Schizophrenia patients. Scores closer to 1 represent a higher probability of being selected for decisions by the Support Vector Machine algorithm. Only features with a selection probability >0.5 are shown (complete selection probability results for the whole pool of cognitive and socio-cognitive features are available in SF2A and 2B, respectively). FEIT = Facial Emotion Identification Test; N = number; RAVLT = Rey Auditory Verbal Learning Test; SEM. FLUENCY = Semantic Fluency; TASIT II/III = The Awareness of Social Inference Test – Section II/Section III; WMS = Wechsler Memory Scale.

Further investigations of the cognitive classifier revealed that the features that mostly contributed to HC2 vs. SCZ discrimination were the 0-back accuracy, the global memory IQ score, the total score of immediate recall for verbal learning^40,41^, the total score for semantic fluency^41^ and 1-back efficiency, computed as the ratio between accuracy and reaction times (Fig. 5). With regard to the socio-cognitive classifier, the most relevant features for the HC2 vs. SCZ classification were the TASIT total score of ability to infer lie, global sarcasm, and paradoxical sarcasm, the FEIT accuracy in identifying surprise facial expressions, as well as the FEIT total average response time (Fig. 5).

#### Phase 2: reciprocal validation of the models discriminating between healthy controls and patients

To investigate whether the disease-related signatures built independently for each cohort (i.e., HC1 vs. BD and HC2 vs. SCZ) were disease-specific or able to correctly categorize individuals of the other cohort, we employed an OOCV to apply HC1 vs. BD discriminative models to the HC2-SCZ cohort, and vice-versa (Methods, “Phase 2: reciprocal validation of the generated unimodal and multimodal classifiers”), as already done previously^42^. OOCV is a technique used when discovery models previously generated by an algorithm are applied to a new set of data collected on independent groups of unseen individuals, with the aim to test signatures’ generalizability^31^. The application of the models discriminating HC1 vs. BD to the HC2-SCZ cohort revealed that all unimodal and multimodal classifiers assigned group memberships within the HC2-SCZ cohort better than chance, with the highest validation performance for the cognitive classifier (BAC: 78.3%), followed by the stacking-based model (BAC: 77.4%) and the socio-cognitive classifier (BAC: 66.2%) (for detailed classification results, Table 4A).

**Table 4:** Reversal discovery-validation strategy classification performance.

	True negatives	True positives	False negatives	False positives	Sensitivity	Specificity	Balanced accuracy (%)	Area under the curve	Positive predictive value	Negative predictive value	Positive likelihood ratio
Classification: Application of HC1 vs. BD generated models to HC2-SCZ cohort											
Cognitive classifier	165	85	33	30	72.03	84.6	78.3	0.85	73.9	83.3	4.68
Socio-cognitive classifier	146	68	50	49	57.6	74.9	66.2	0.73	58.1	74.5	2.29
Stacking-based multimodalclassifier	158	87	31	37	73.7	81.02	77.4	0.85	70.2	83.6	3.99
Classification: Application of HC2 vs. SCZ generated models to HC1-BD cohort											
Cognitive classifier	89	42	17	6	71.2	93.7	82.4	0.88	87.5	83.9	11.3
Socio-cognitive classifier	82	41	18	13	69.5	86.3	77.9	0.81	75.9	82	5.07
Stacking-based multimodal classifiers	87	44	15	8	74.6	91.6	83.1	0.88	84.6	85.3	8.85

A: Classification performance of the models discriminating Healthy Controls (group 1) vs. Bipolar Disorder patients and applied to the data of the cohort including Healthy Controls (group 2) and Schizophrenia patients without any in-between retraining. B: Classification performance of the models discriminating Healthy Controls (group 2) vs. Schizophrenia patients and applied to the data of the cohort including Healthy Controls (group 1) and Bipolar Disorder patients without any in-between retraining.

*BD* patients with Bipolar Disorder; *HC1* Healthy Controls (group 1); *HC2* Healthy Controls (group 2); *SCZ* patients with Schizophrenia.

Applying the models discriminating HC2 vs. SCZ to the HC1-BD cohort indicated that both unimodal and multimodal classifiers assigned group memberships within the HC1-BD cohort better than chance, with the stacking-based model as the best-performing classifier (BAC: 83.1%), followed by the cognitive (BAC: 82.4%) and the socio-cognitive classifier (BAC: 77.9%) (for detailed classification results, Table 4B).

For supplementary models aimed at classifying BD and SCZ using a direct comparison of the two groups of patients, please see Supplementary Information - SI, Section 5.

#### Phase 3: application of the multimodal classifiers discriminating between healthy controls and patients to First Episode of Psychosis and Clinical High-Risk individuals

**Fig. 6:**
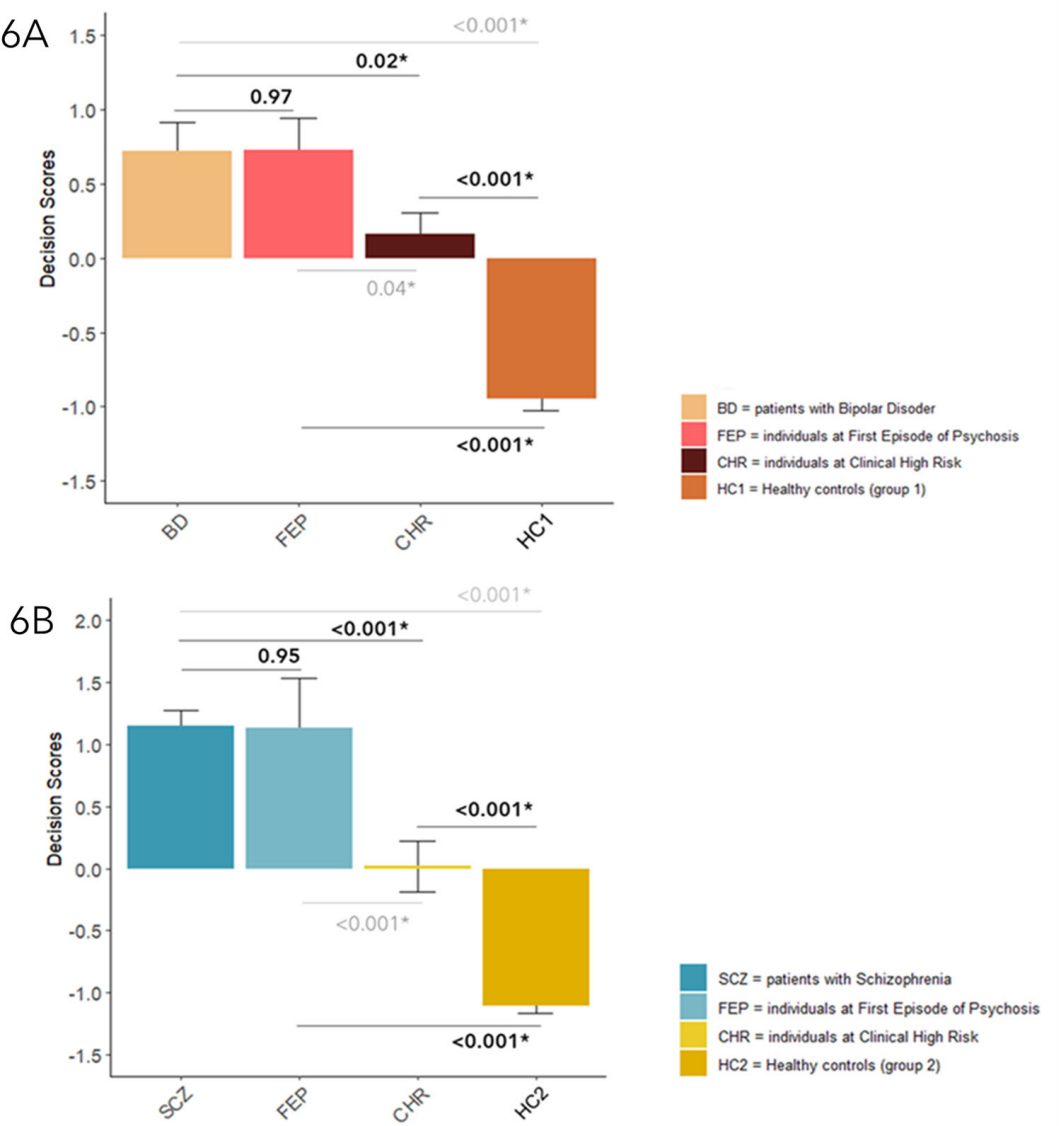
Between-groups comparisons. ANOVA analysis were conducted to compare decision scores from the stacking-based models discriminating Healthy Controls (group 1) vs. Bipolar Disorder patients (panel 6A) and Healthy Controls (group 2) vs. Schizophrenia patients (panel 6B) and decision scores extracted for Clinical High Risk and First Episode of Psychosis individuals after the Out-Of-sample-Cross-Validation procedure. Error bars represent standard error.

Then, we explored the possible generalization of the best-performing HC1 vs. BD and HC2 vs. SCZ classifiers (i.e., stacking-based) to earlier stages of disease and psychosis risk. Specifically, we first applied both the disease-related stacking-based models to CHR and FEP cognitive and socio-cognitive data via OOCV. Then, we used decision scores based on such classifiers as the dependent variable in an ANOVA model (between factor: membership to HC1/HC2, CHR, FEP, BD or SCZ groups) (see Methods, “Phase 3: investigating the generalization of unimodal and multimodal classifiers” for details). The application of the HC1 vs. BD model revealed that decision scores for FEP significantly differed from those for HC1 (*p* = <0.001), but not from those for BD (*p* = 0.97), while CHR decision scores differed from both those for HC1 (*p* < 0.001) and for BD (*p* = 0.02) (Fig. 6A). When the HC2 vs. SCZ model was applied, FEP decision scores were different from those for HC2 (*p* < 0.001), but not from those for SCZ (*p* = 0.95). Differently, CHR decision scores differed from both those for SCZ (*p* < 0.001) and for HC2 (*p* < 0.001) (Fig. 6B). In summary, after the application of the HC1 vs. BD and HC2 vs. SCZ stacking models, FEP individuals showed both a BD- and an SCZ-like classification pattern, whereas neither a control nor a patient-like pattern emerged for CHR individuals.

## Discussion

Our results suggest that cognitive and socio-cognitive deficits are relevant to the multimodal categorization of SCZ or BD vs. HC, and that anomalies contributing to such discrimination are mostly disease-specific, with some overlap. On the other hand, multivariate models taking into account the entire set of these cognitive and socio-cognitive impairments are not predictive for differentiation between patients with these two brain disorders. Furthermore, the present findings also suggest a generalization of cognitive and socio-cognitive classifiers categorizing SCZ and BD vs. HC only to first episode of the illness, but not to conditions of clinical liability.

### Classification models between healthy controls and patients (Phase 1)

We found that unimodal models based on either cognitive or socio-cognitive characteristics significantly and accurately assigned membership of BD and SCZ to the respective diagnostic groups. Indeed, the combination of these unimodal classifiers in a single stacking model led to the highest discriminative power between classes. These results suggest that cognitive and socio-cognitive profiles of alterations contribute to the phenotypical characterization of schizophrenia and bipolar disorder^23^.

Analysis of the cognitive and socio-cognitive features contributing the most to the discrimination between BD or SCZ from the respective HC cohorts allows to further characterize this contention. In particular, the most reliable cognitive features for the discrimination of BD were related to overall working memory accuracy and global memory quotient, suggesting a memory-related pattern of alterations consisting of load-independent working memory impairments and multi-domain memory disruptions at the core of the disorder. On the other hand, disruptions within the domain of memory, namely the global memory quotient and load-dependent working memory efficiency, contributed to discriminate SCZ from HC, together with other cognitive deficits related to information processing (0-back accuracy), incidental immediate learning, and semantic fluency. Therefore, a broader pattern of multi-domain cognitive anomalies, already reported by previous literature^10,19^ and mainly related to basic information processing and verbal-executive components of cognition, emerged for schizophrenia. Taken together, these results suggest that cognitive abnormalities differentiating either BD or SCZ from HC are generally qualitatively different, but commonalities are also present. Interestingly, findings from univariate literature primarily implicate quantitative rather than qualitative differences in cognitive anomalies between BD and SCZ^10,43^, which may be consistent with the view that a data-driven, multivariate approach is more sensitive to a fine-grained disentanglement of the cognitive asset of these two disorders.

Our results from the socio-cognitive domain may be inserted in a similar explanatory framework. We found that the socio-cognitive variables contributing the most to BD discrimination were the accuracy in identifying angry facial expressions and the global time needed to identify facial emotion expressions. These results appear to delineate a pattern of alterations mainly related to emotion identification processes and are partially consistent with previous univariate literature reporting slower reaction times in BD during emotion recognition^44^ and decreased accuracy in identifying^45^ and distinguishing^46^ anger from other emotions^44^^,^^47^. Results in SCZ indicate that the most reliable features selected to classify between patients and controls were the ability to properly infer both lie and global sarcasm, the ability in identifying surprise facial expressions, and the average response time to correctly identify emotions on faces. Previous literature already reported deficits in enriched social inference in schizophrenia. In particular, a previous study indicated lower performance in SCZ at both lie and sarcasm TASIT subscales when compared with BD and HC individuals, with the latter two groups having similar performance^23^. Similarly, surprise was already reported as the most misinterpreted emotion by SCZ patients, although within a frame of disrupted emotion recognition^48^.

Overall, these findings suggest that socio-cognitive features reveal a key discriminative power in discriminating BD or SCZ from HC, which is mostly related to the emotion recognition and social inference broader domains, consistently with previous literature reporting their alteration in both these brain disorders^11^. Furthermore, as for the cognitive domain discussed above, our findings also suggest both qualitative differences and overlaps in discriminant socio-cognitive features in BD and SCZ vs. HC. Specifically, a broad spectrum of socio-cognitive anomalies, encompassing basic facial emotion identification and both minimal and enriched social inference, emerged for schizophrenia. Differently, BD were discriminated from HC by a prevalent occurrence of emotion identification deficits. A possible interpretation of these results is that SCZ are discriminated by alterations of complex social processes as well as by minimal social inference abilities, while discrimination of BD is mainly supported by the latter. However, it should be noted that also the ability to properly infer paradoxical sarcasm was highly discriminant for classifying both BD and SCZ. Sarcasm comprehension has been previously associated with ToM abilities^49^ and impairments in this social ability are reported for both disorders, although a greater deficit appears to be present in schizophrenia^50^. Thus, we may speculate that ToM deficits could trans-diagnostically affect the ability to make judgments on the meaning of conversational remarks both in schizophrenia and bipolar disorder. However, hierarchical models able to better specify the reciprocal relationships among paradoxical sarcasm and ToM abilities need to be developed in the future to validate this view.

### Reciprocal validation of the models discriminating between healthy controls and patients (Phase 2)

Results from the between-cohort application of within-cohort generated models indicate high performance of classification for the cognitive, the socio-cognitive, and the stacking-based classifiers, with high discriminative power of all the HC1 vs. BD generated models in the HC2 vs. SCZ comparison, and vice-versa. These results suggest that the overall signatures generated for each disorder are not disease-specific. On the other hand, we found that some of the cognitive and socio-cognitive features discriminating the most between BD vs. HC1 and SCZ vs. HC2 do not overlap, which seems at odds with the generalizability of the overall models between diagnoses. A possible explanation of these seemingly discrepant findings is that the variables selected as most reliably discriminating between HC1 and BD, and between HC2 and SCZ, respectively, were different because our ML pipeline identified deficits that are more “at the core” of each disease at the single-subject level, and therefore representing “hub” cognitive and socio-cognitive diagnosis-related deficits. Despite the identification of these “core”, diagnosis-related alterations, the high generalization of each model to unseen individuals with a different diagnosis proved the non-specificity of the overall bipolar and schizophrenia signatures, suggesting how, at the level of the entire pool of variables, our algorithm may have caught further cognitive and socio-cognitive deficits relevant for both the disorders. Based on this interpretation, our results may support the notion that schizophrenia and bipolar patients are characterized by similar cognitive and socio-cognitive deficits, consistently with previous univariate literature^10,11^. However, within these deficits, those that are more at the core of each disease are different. Therefore, personalized programs targeted at chronic patients should be oriented to primarily manage disease-related “hub” alterations, but always within a broader framework of intervention aimed to monitor also to the “side”, less central, trans-diagnostic deficits. According to this interpretation, our findings might be considered as a starting evidence corroborating the existence of a psychopathological continuum between schizophrenia and bipolar disorder, emerging at the level of cognitive-behavioral phenotypes. However, as widely reported by previous literature conceptualizing bipolar disorder and schizophrenia as different brain diseases^51^, cognitive and socio-cognitive deficits in both the disorders might be underpinned by multiple structural and functional abnormalities^52,53^. Thus, delivering classification models based also on brain features is needed to more deeply investigate—and potentially corroborate—the bipolar disorder-schizophrenia continuum hypothesis. For instance, to build new knowledge useful to better refine current nosology, future machine learning studies generating models based on both behavioral and structural/functional brain data should aim at combining this multivariate information to grasp the possible between-domains latent interactions at the single-subject level.

### Application of the multimodal classifiers discriminating between healthy controls and patients to First Episode of Psychosis and Clinical High-Risk individuals (Phase 3)

As a final step, we tested the generalization of each stacking-based signature (i.e., HC1 vs. BD and HC2 vs. SCZ) to earlier stages of diseases and to psychosis risk. Here, FEP decision scores did not differ from those of BD or SCZ when the respective predictive models were applied, while such scores diverged from those of HC individuals. These patterns indicate that, for both HC1 vs. BD and HC2 vs. SCZ models, the algorithm recognizes FEP more as “BD-like” or “SCZ-like”, rather than “HC-like”, suggesting that both BD-related and SCZ-related multimodal signatures generalized to early stages of disease irrespectively from diagnostic boundaries, thus showing trans-diagnostical prognostic relevance.

A different pattern of results was present when both HC1 vs. BD and HC2 vs. SCZ models were separately applied to CHR. Here, CHR decision scores differed from both those of BD and SCZ as well as from those of HC individuals. In other words, for both HC1 vs. BD and HC2 vs. SCZ models, neither patient- nor control-like patterns emerged for CHR, suggesting that cognitive and socio-cognitive features of individuals at clinical risk diverge from those of HC, consistently with the notion that these individuals may be affected by anomalies related to these domains; however, such cognitive and socio-cognitive impairments may not be shaped as those present in full-blown schizophrenia or bipolar disorder yet.

#### Limitations

Our results should be interpreted considering several limitations. Although we carefully controlled for potential confounding factors including age and sex (see SI, Section 4 for further details about ML preprocessing pipeline), their nonlinear interaction with other external confounds, like medication itself, onset of disease and duration of illness or premorbid intelligence quotient, cannot be ultimately ruled out. However, considering that no significant associations emerged between decision scores extracted from our best models and psychopharmacological treatment or symptoms, it is less likely that these confounders might have affected our results. Moreover, because of the lack of information about non-pharmacological interventions that patients may have undertaken, we could not explore their putative effects on cognitive and socio-cognitive performance. Future studies are warranted to shed light on this possibility.

Although we have employed a stringent double cycle nested Cross-Validation (CV) strategy which should enforce unbiased estimations, further validations of our results in external wider groups of individuals with completely different demographic and geographic profiles are needed to fulfil requirements for generalizability. Indeed, this further evidence would strengthen the potential for translation of our findings into clinical practice and is particularly warranted for the at-risk and FEP cohorts, whereas validation results may have been affected by the small sample size.

#### Conclusions and future directions

We delivered ML algorithms highlighting that, despite “hub” cognitive and socio-cognitive alterations specifically related to each full-blown diagnosis can be identified, bipolar disorder and schizophrenia share in both domains an overall common pattern of impairments that should therefore be trans-diagnostically approached. Therefore, effective remediation strategies for BD or SCZ individuals should be tailored both on these specific cognitive and socio-cognitive deficits at the core of each disorder, and on less central behavioral alterations. Furthermore, our findings support the potential translation of such trans-diagnostic intervention strategies at earlier stages of diseases (i.e., first-episode individuals). In this framework, we think our results are potentially relevant from a clinical perspective, as they provide ready-to-use information to refine individualized intervention focused on cognitive and socio-cognitive impairments for both the earlier and the chronic phases of the diseases^32^. Nevertheless, future studies are warranted to further validate our findings and investigate more deeply the relationship between the identified cognitive and socio-cognitive alterations from a trans-diagnostic perspective as well as their potential associations with brain structural and functional underpinnings.

## Methods

### Sample determination

A total of 546 individuals, all Caucasians native of the Apulia region, Italy, participated in the study. Inclusion and exclusion criteria and full details about sample determination are reported in SII, Section 1. Specifically, our sample included 290 HC and 177 patients, of which 118 were SCZ and 59 were BD, according to the Diagnostic and Statistical Manual of Mental Diseases-5 criteria^3^. To obtain two cohorts with a proportion between healthy subjects and patients of about 2:1, 95 HC (Healthy Controls - group 1: HC1) randomly entered the first cohort with the BD group (Table 1A), while the remaining 195 HC (Healthy Controls - group 2: HC2) entered the second cohort with the SCZ group (Table 1B).

Moreover, individuals at risk for psychosis or at early stages of disease were also included (Table 2). Specifically, 35 were CHR individuals (i.e., at clinical risk for a first episode of psychosis^54,55^) and 29 were FEP individuals (i.e., individuals at first episode of psychosis). A detailed description of the clinical characteristics of these cohorts is reported in SI, Section 1.

For all participants, we assessed socio-economic status with the Hollingshead scale^56^ and the Intelligence Quotient (IQ) using the Wechsler Adult Intelligence Scale-Revised (WAIS-R)^57^. Furthermore, the Italian version of the Wide Reading Achievement Test (WRAT)^58^ was employed to measure premorbid IQ. In clinical populations, the Global Assessment of Functioning Scale (GAF)^59^, the PANSS, and the YMRS were administered to evaluate patients’ global functioning, positive and negative symptoms, global psychiatric symptoms, and manic symptoms.

Two sample t-tests, χ^2^ tests, and ANOVA were used to investigate group differences in terms of demographic and clinical characteristics, both within (Table 1A, B) and between cohorts (Table 2).

### Machine Learning: definition of unimodal classifiers

All participants underwent an extensive assessment protocol, aimed at collecting data about cognitive and socio-cognitive abilities in adult healthy and clinical populations, through different standardized tools (SI, Sections 2–3). Using these cognitive and socio-cognitive measures, we trained:

- a “cognitive” classifier based on variables from different target cognitive domains previously associated with bipolar disorder^2^ and schizophrenia^60^. Specifically, we fed the algorithm with 52 variables, reflecting individual abilities based on psychometric norms from different pen-and-pencil neuropsychological tools, capturing attention (AX Continuous Performance Task – CPT^61^, TMT – Part A and B), global memory (WMS), incidental (RAVLT) and episodic (Babcock Story Recall Test - BSRT^62,63^) auditory verbal learning, working memory (N-back), verbal fluency (Phonological and Semantic Fluency Test) and abstract reasoning (Wisconsin Card Sorting Test – WCST^64^) performance.
- a “socio-cognitive” classifier based on variables from three social cognition domains of interest, i.e., identification of emotions, social inference, and emotion management, reported as frequently altered in both schizophrenia and bipolar disorder^65^. In particular, the algorithm was fed by 37 variables, which included domains measured with: (1) the FEIT, which allowed quantifying the individual ability to correctly identify and process emotions from facial expression; (2) the TASIT, which measures the higher-level ability to infer other people’s emotions and thoughts using contextual information during social interactions; the Mayer-Salovey-Caruso Emotional Intelligence Test (MSCEIT)^66^ which measured the ability to efficiently manage emotional reactions during fictional every-day situations.

SI reports detailed descriptions of the tools administered (Sections 3 and 4), a complete list of the variables which fed ML models (Supplementary Table-ST1), as well as mean and standard deviation performance values of each of these variables (ST2A and ST2B).

### Machine Learning: analysis pipeline

Our overall ML analytic strategy consisted of three phases and was carried out using the NeuroMiner software, version 1.05 (www.pronia.eu/neurominer/)^30^. In particular, to properly detect cognitive and socio-cognitive similarities and differences between schizophrenia and bipolar disorder, we employed a “reversal discovery-validation strategy”, as already done in previous publications^42^, consisting in two phases, i.e., Phases 1 and 2. In a third step (Phase 3), we investigated how discriminative models generated and validated in the previous phases applied to patients at early stages of disease and at-risk populations.

#### Phase 1: generation of unimodal and multimodal classifiers

We first generated parsimonious unimodal and multimodal classification models based on our cognitive and socio-cognitive domains of variables to build disease-related signatures, that could discriminate BD and SCZ clinical groups from HC1 and HC2, respectively (see Results, “Phase 1: classification models between Healthy Controls (group 1) and Bipolar Disorder patients” and “Phase 1: classification models between Healthy Controls (group 2) and Schizophrenia patients”).

In our ML pipeline (full details in SI, Section 4) we implemented a double-cycle, repeated nested CV^30,42,67^, to allow for unbiased estimation of the model’s generalizability, preventing information leaking throughout the strict separation between subjects used for training the models and independent individuals used for testing decisions^68^. According to this strategy, models trained at the inner CV cycle (CV1) of each unimodal classifier (i.e., cognitive and socio-cognitive) that contributed most to the discriminative pattern between HC1 vs. BD and HC2 vs. SCZ, were then applied to independent test data at the outer CV cycle (CV2). We implemented a 10-fold CV cycle both in the inner CV1 and in the outer CV2 levels, employing a repeated nested CV^29^. In particular, we permuted the participants within their groups (number of permutations = 5) and repeated the CV cycle for each of these permutations. Thus, within our nested CV framework, we built an ensemble of 50 models (*n* = 5 repetition × *k* = 10 folds) for each CV2 partition^30,31^. All model training steps of parameter optimization, that use group-level statistical procedures for features’ preprocessing, occur only in CV1 training data. Instead, CV1 test data are used to pick hyperparameter combinations that provide potentially good model generalization capacity. Specifically, all the features entering our classifiers underwent three preprocessing steps within CV1:

i. feature-wise scaling was performed to remove between-features differences effect from the training sample information;
ii. scaled data entered a k-Nearest Neighbor imputation step, aimed at filling the missing values for each given subject in the data, using the feature-specific median value computed in the 7 more similar nearest neighbors on the basis of Euclidean distance;
iii. partial correlations were finally performed to regress out the variance associated with age and gender and control for their confounding effect, given the reported association between these variables and the heterogeneity and severity of cognitive^69,70^ and socio-cognitive^71,72^ alterations in schizophrenia and bipolar disorder (see SI-Section 4.3 for details).

To assess the discriminative utility of the input variables within each unimodal classifier, we performed a feature selection procedure within the CV1 loop^29,42,73^. More specifically, after the data entering in a greedy forward search wrapper^73^, we computed the feature-related probability of being selected for classification purposes within the inner CV loop for each variable, providing maximum prognostic performance with the smallest amount of predictors. This way, we then applied the trained model to CV2 cycle, where generalization error estimation is performed^68^. Indeed, CV2 validation data, as derived from unseen study participants (i.e., they were not used for training the classification algorithm)^68^, serve exclusively the purpose to measure the models’ generalizability. With this procedure, we finally obtained parsimonious SVM decision models based on preprocessed cognitive and socio-cognitive features, determining each validation individual’s outcome class (i.e., HC1/HC2 vs. BD/SCZ) through a majority voting procedure across all ensemble models. Classification performance was measured using BAC, sensitivity, specificity, Area-Under-the Curve, Positive Predictive Value, Negative Predictive Value, and Positive Likelihood Ratio. A stacking procedure was further implemented to build a third multimodal classifier^74^, aimed at combining the unimodal classifiers within the ML environment. Specifically, stacking used the decisions models from our lower-level cognitive and socio-cognitive unimodal classifiers to generate a new, higher-level algorithm that did not learn from domain-specific raw data (i.e., cognition and social cognition performance scores), but from the decisions scores of the unimodal classifiers^29^. The stacking generalization was performed to investigate whether using all the information coming from both cognition and social cognition would have led to an increase in HC1 vs. BD and HC2 vs. SCZ classification accuracy, compared with the classification ability of single domains-related unimodal classifiers. Permutation analyses were performed to assign statistical significance to the observed classification performance of our models^30,75^. Specifically, for each permutation, we performed 1000 random permutations of the outcome labels and we retrained all linear SVM models in the repeated nested CV design, using the respective feature subsets resulted from the observed-label analyses. For each permutation, we collected the predictions of the random models into a permuted prediction ensemble for each outer cycle subject. Thus, we built a BAC-based null distribution of out-of-training classification performance. The final calculated significance of the observed out-of-training BAC consisted in the number of events with a permuted out-of-training BAC higher or equal to the observed BAC divided by the number of performed permutations. We set the model significance at *α* = 0.05. To explore a possible relationship between classification and medication or symptoms severity, we performed non-parametric correlation analyses between decision scores extracted from the best-performing classifiers in both the cohorts and drugs dosages, scores of mania (as assessed by YMRS) and psychosis-related psychopathology (as assessed by PANSS) (*α* = 0.05, not corrected).

#### Phase 2: reciprocal validation of the generated unimodal and multimodal classifiers

We applied (i) HC1 vs. BD models to the HC2-SCZ cohort, and vice-versa, (ii) HC2 vs. SCZ models to the HC1-BD cohort using OOCV^31,33^, without any re-in-between training. Specifically, OOCV is usually required in an ML framework for a thorough generalizability assessment and usually involves the application of the trained models used to predict targets in the CV2 data folds (i.e., HC1 vs. BD and HC2 vs. SCZ models) to a separate dataset that has been independently collected (i.e., HC2-SCZ and HC1-BD data, respectively). Thus, this method allowed us to investigate whether the disease-related signatures built independently for each cohort were disease-specific or had discriminative power also for the other (see Results, “Phase 2: reciprocal validation of the models discriminating between healthy controls and patients”).

#### Phase 3: investigating the generalization of unimodal and multimodal classifiers

To explore the generalization of the independently generated HC1 vs. BD and HC2 vs. SCZ ML signatures to earlier stages of diseases and to psychosis risk, we compared the performance of the best classifier in assigning membership to HC or patient groups with those obtained applying such classifier to CHR and FEP. With this aim, we performed an OOCV without any in-between retraining as done in Phase 2 (see Methods, “Phase 2: reciprocal validation of the generated unimodal and multimodal classifiers”). Then, we employed an ANOVA to compare the OOCV-based ML decision scores of each of these cohorts, with, respectively, the HC1 vs. BD and the HC2 vs. SCZ decision scores (see Results, “Phase 3: application of the multimodal classifiers discriminating between healthy controls and patients to First Episode of Psychosis and Clinical High-Risk individuals”). Therefore, in terms of decision scores comparison, the presence of a significant difference between CHR or FEP and any other clinical group would suggest that the OOCV-ed algorithm did not classify them as patients. On the other hand, the presence of a significant difference between CHR or FEP and any control group would suggest that the algorithm did not classify them as controls. Finally, the presence of significant differences between CHR or FEP and both controls and clinical cohorts would suggest that CHR and FEP individuals were not comparable either to controls or to patients, based on the rule generated by the OOCV-ed algorithm.

## Supplementary information

Supplementary Information
