# Supplementary material for: Similarities and differences between multivariate patterns of cognitive and socio-cognitive deficits in schizophrenia, bipolar disorder and related risk
Source: Schizophrenia (Heidelb). 2023 Feb 17;9(1):11. doi: 10.1038/s41537-023-00337-0 (PMC9938280; doi:10.1038/s41537-023-00337-0)
Supplement: Supplementary file 1 — Supplementary Information [file 41537_2023_337_MOESM1_ESM.docx]

**SUPPLEMENTARY INFORMATION**

**1. Sample determination**

A total of 546 individuals, all Caucasians native of the Apulia region, Italy, participated in the study. Specifically, our sample included 290 healthy controls, randomly split in two independent groups (Healthy Controls - group 1: HC1; Healthy Controls - group 2: HC2), and 177 patients, of which 118 were diagnosed with schizophrenia (SCZ) and 59 with bipolar disorder (BD).

SCZ or BD diagnoses were assessed with the Structured Clinical Interview for DSM-IV-TR (SCID)^1^. At the moment of the study inclusion, both selected SCZ and BD were on stable pharmacological treatment for at least one month.

Moreover, 79 individuals at different early stages of psychosis risk and at the initial stage of disease were also included. Of those, 35 were identified as Clinical High Risk (CHR) (i.e., at enhanced risk for development of a first-episode psychotic disorder^2^) and 29 were First Episode of Psychosis (FEP) individuals.

The CHR state was defined by: (1) cognitive disturbances, as assessed by the Schizophrenia Proneness Instrument (SPI-A)^3^, and/or (2) Ultra-High-Risk criteria for psychosis, according to the Structured Interview for Psychosis-Risk Syndromes criteria (SIPS)^4^. CHR were excluded when they had an intake of antipsychotic medication for more than 30 cumulative days, and when they had any intake of antipsychotic medication within the past 3 months before study enrollment^5^. Individuals were identified as FEP when they met criteria for not more than one Diagnostic and Statistical Manual for Mental Disorders-5^6^ affective or non-affective psychotic episode fulfilled within the past 3 months, and when the onset of psychosis happened within the past 24 months^5^. FEP were excluded when they underwent antipsychotic medication for more than 90 days (cumulative number of days)^5^.

All participants had no history of drug or alcohol abuse within the last 6 months, head trauma with loss of consciousness, or any other clinically significant medical condition.

All the participants who underwent psychiatric and neuropsychological assessment procedures were mental healthcare professionals at the Bari University Hospital (Psychiatric Unit) or at the enrollment sites included in the Apulian Network on Risk for Psychosis. Specifically, bipolar disorder, schizophrenia, first episode of psychosis patients, and clinical high-risk individuals were diagnosed by experienced psychiatrists, specifically trained to properly administer all the scales of clinical assessment. All the cognitive and socio-cognitive assessment tools were administered by clinical psychologists with certified expertise in neuropsychology.

All individuals enrolled in the study were recruited at the University of Bari Aldo Moro (Italy), after the approval of the protocol by the local research ethic committee and provided their written informed consent before study inclusion. All procedures performed were in accordance with the 2013 Helsinki Declaration or comparable ethical standards.

**2. Cognitive classifier composition: assessment of neuropsychological abilities**

All individuals were administered eight tests, each aimed at investigating a different aspect of neuro-cognitive abilities across six different domains (a complete list of the administered tests with corresponding scores that entered both the neuro- and the socio-cognitive classifier is reported in Supplementary Table 1 – ST1):

a. to investigate the attention domain, we employed a computerized version of the AX – Continuous Performance Task (CPT)^7^ and a pen-and-pencil version of the Trail Making Test (TMT)^8, 9^.

- The **CPT** was developed to measure sustained and selective attention, but through the AX version is possible to measure goal representation, maintenance and updating^10^ too. During the task, single yellow letters were presented sequentially on a black screen, at the speed of about 1 second per letter. Participants are required to answer, using the space bar on a keyboard, when they see a probe X letter following a cue A letter. Randomly, different other conditions can follow each other during the 15 minutes of administration: a non-cue letter could precede the probe (e.g., B-X), the cue itself could be followed by a probe-like or non-like distractor (e.g., A-Y or A-D), the non-cure letter could precede a probe-like or non-like distractor (e.g., B-Y or B-D). The manipulation of the expectancy of the cue A letter and the probe X letter (70% of the trials) creates two distinct conflictual situations. In AY or AD trials, the participant expects to see an X after the A and must inhibit the prepotent response when facing a non-X letter, with more inhibition effort if the Y probe-like distractor appears. In BX trials, the X probe triggers an automatic response that must be inhibited since the cue is B as a non-A letter. In BY or BD trials, the participant needed to continue to inhibit the response since the cue is B as a non-A letter, with more inhibition effort if the Y probe-like distractor appears.

- The **TMT** is a handy pen-and-pencil task, effective in the assessment of multiple measures of processing speed, selective and divided attention, and mental flexibility. The original version included two parts: on part A, examinees are required to connect, in ascending order and trying to never detach the pen from the paper, 25 encircled numbers randomly placed on a page in a predefined order; on part B, encircled numbers from 1 to 12 are randomly placed on the page, mixed with encircled letters from A to L: in this second part, the request for the examinees is to connect circles alternating numbers and letters, following ascending and alphabetic orders respectively. The test is scored based on the time, in seconds, to complete the task^11^, so that higher time to complete Part A might highlight possible abnormalities in processing speed and selective attention processes, whereas low scores on part B may suggest difficulty in the more complex component of the executive-attentive functioning, like divided attention and flexibility in the shifting between different sets of stimuli. Errors are not counted in the time score other than the addition of time to go back to the previous error and continue on^11^.

b. to investigate the language domain, we employed the Italian version of the Verbal Fluency test ^12-14^.

- The **Verbal Fluency** test was basically developed to assess the ability to access the verbal lexicon, retrieving specific information within restricted search parameters^11^, that can be related both to phonological and to semantic rules. Therefore, our paper-and-pencil version of the Phonological Fluency test required examinees to generate words beginning with letters F, A, and S, in a limited timeframe of 1 minute per letter. Similarly, our paper-and-pencil Semantic Fluency test asked the examinees to generate words belonging to three semantic categories, including colors, fruits, and animals, always with 1 minute left per category. The two final Phonological and Semantic Fluency scores derive from the sum of all generated words among the three phonemic and semantic categories respectively. Because Verbal Fluency is a cognitive function that facilitates information retrieval from memory^11^, both Phonological and Semantic Fluency tests don’t examine only language abilities only, but require and assess the involvement of further higher-level executive processes such as selective attention, selective inhibition, mental set shifting, internal response generation, and self-monitoring^11^.

c. to investigate the memory domain, we employed the Wechsler Memory Scale – Form III (WMS-III)^15^, the Babcock Story Recall Test (BSRT)^16, 17^ and the Rey Auditory Verbal Learning Test (RAVLT)^12, 13, 18^ in their Italian, pen-and-pencil version.

- The **WMS** is the original Wechsler Memory Scale developed by David Wechsler in 1945^19^ to provide a global assessment of mnestic functioning. Through the complete administration of the scale (we administered the third version), a composite Memory Quotient can be computed from seven sub-tests and their related indexes, which covered all the main possible memory domains.

The first and second sub-tests, the subject is asked to answer some simple questions about personal and general basic knowledge as well as about spatial-temporal orientation.

The third subtest included three simple time trials (i.e., reversal and three from three countings, repetition of alphabetic letters) aimed at evaluating mental control abilities during the manipulation of verbal and numeric stimuli, with the possibility of calibrating the score attribution for each item consistently with the number of committed errors.

In the fourth subtest, aimed at evaluating declarative episodic memory, the subject is asked to hear two brief stories and, after listening to each one, to recall as many elements as possible. Each item recalled is given one point as long as it is reproduced by the subject using the same words as the administer, while the total score of the subtest is given by the sum of the items correctly recalled for each story divided by 2.

In the fifth subtest, verbal short-term and working memory is assessed, throughout a Digit Span subtest comprehensive of two parts: the digits forward, where the examinee is asked to repeat increasing spans of digits in the order they were presented; and digits backward, where the examinee is asked to repeat increasing spans of digits in reverse order^11^. Two trials are administered for each span length in both conditions, whereas both the forward and the backward are discontinued after the failure of both trials for the same span^11^.

In the sixth subtest, aimed at evaluating visual short-term memory, the subject is asked to observe four complex geometric figures, of which the first two are shown one after the other and the last two together, for a total of three trials lasting respectively 10, 10, and 30 seconds. After the time expires for each trial, the administer covers the figure shown and the examinee is asked to reproduce it, trying to recall as many elements as possible. The global score for the subtest is computed from the single trials recall scores, determined by the number of elements recalled for each figure and their correct placement in the figure structure.

The last, seventh subtest is aimed at assessing verbal associative learning: this list-learning paradigm included three independent following trials, where the subject is asked to hear 10 pairs of words. The number of words recalled during each of these five repetitions provides a measure of immediate memory span. The change in the number of recalled words over these five trials provides a learning curve, with the slope of the curve representing the process of verbal auditory learning and demonstrating the relative memory capacity.

- The **BSRT** was developed to assess episodic verbal immediate and delayed long-term memory. During the administration, the examinee is asked to hear a short story, which, in our version, consists of 4 main elements to code and recall and 5 secondary elements. After the first reading from the examiner, the examinee is asked to recall as many elements as possible from the story and, once finished, to hear a second reading of the same story, but without any immediate recall after this further listening: indeed, the subject will be asked to perform a delayed recall 10 minutes later. The final global score derives from the sum of the total number of elements reported by the examinee during both the immediate and the delayed recall, within a hierarchical scoring system where some secondary elements can be included in the score computation only if related main elements were recalled too.

- The **RAVLT** was developed to evaluate verbal auditory incidental memory and can be used both to track changes in memory function over time and to compare specific memory processes within the same individual^11^. The test consists of a list-learning paradigm where, for five repetitions, the patient hears 15 semantically unrelated nouns (read by the administer one after another at a rate of one name per second) and is asked to recall as many words from the list as possible, not necessarily in the same order of presentation. The number of words recalled during each of these five repetitions provides a measure of immediate memory span^11^. The change in the number of recalled words over these five trials provides a learning curve, with the slope of the curve representing the process of verbal auditory learning and demonstrating the relative memory capacity^11^. After a delay of 20 minutes from these five repetitions of free recall, the participant performs a “delayed recall” task, where is asked to again recall the words from the list learned 20 minutes before. The delayed recall score compared to the number of words recalled on the trial one asses the participant’s retention skill^11^.

d. to investigate the working memory domain, we employed a computerized version of the N-Back task^20^.

- The **N-Back task** was developed to properly assess working memory, as a form of short-term memory that enables actively holding multiple pieces of information in mind and manipulating them^21^. In our computerized version of this task, participants were asked to complete a blocked paradigm of the N-back: numbers from 1 to 4 were shown in random sequence on a computer screen and displayed in four corners of a diamond-shaped box. In the 0-back, i.e., the non-memory-guided condition, examinees were simply asked to identify the currently visible stimulus, by pressing on a keyboard the key corresponding to the number displayed. In the working memory conditions, examinees were required to recollect and answer one or two (1 or 2-back) stimuli seen beforehand while continuing to encode additional incoming stimuli. The complete task consisted of 3 blocks for each condition, each lasting 30 seconds.

e. to investigate the executive domain, we employed the pen-and-pencil version of the Wisconsin Card Sorting Test (WCST)^22^.

- The **WCST** is one of the most common and effective tasks to assess abstract reasoning, i.e., the ability to develop abstract concepts, as well as cognitive flexibility and problem solving, i.e., the ability to shift between different sets^11^. In our paper and pencil format, the subject receives two decks of 64 preordered stimulus cards, with an additional set of 4 reference cards, each differing from the other in terms of 3 categories: color (red, blue, green, or yellow), shape (triangle, circle, square, or cross), or number (1, 2, 3, or 4). The examinees are simply asked to match the stimulus cards to the reference cards, autonomously finding a matching criterion without any further instructions about what it could be. The examiner provides only one simple feedback of ‘‘correct’’ or ‘‘incorrect’’ based on the predetermined category, unknown for the examinees. The subject should be able to use this environmental information to change or proceed with his current criterion in placing the next card^11^. During administration, the rules for the correct categories are changed after the subject correctly matches consecutively 10 cards per category. The WCST provides multiple scores including trials administered, total correct responses, total errors, perseverative responses, perseverative errors, non-perseverative errors, categories completed, failures to maintain set, and a learning-to-learn score^11^.

**3. Socio-cognitive classifier composition: assessment of social cognition abilities**

All individuals were administered three tests, each aimed at investigating a different aspect of socio-cognitive abilities:

a. to investigate emotion perception, we employed the Facial Emotion Identification Test (FEIT)^23^ (details about the Italian version can be found at ^24^); in the computerized FEIT version^25^ we administered, 19 individuals’ faces depicting one of six different emotions (happiness, sadness, anger, surprise, fear, shame) were shown, at a time of 15 seconds each, with 10 seconds of inter-stimulus blank screen during the presentation. 15 photographs depicted negative emotions (sadness, anger, fear, and shame), while 4 photographs depicted positive emotions (happiness, and surprise). After the presentation of each stimulus, individuals were required to mark on an answer form which of the six emotions was depicted on the picture.

b. to investigate theory of mind, we employed The Awareness of Social Inference Test (TASIT)^26^ (details about the Italian version can be found at ^24^). The TASIT consists of seven scales (positive emotions, negative emotions, sincere, simple sarcasm, paradoxical sarcasm, sarcasm enriched, lie), organized into three sections: in the first “emotion recognition” section, 28 videoclips of professional actors enacting ambiguous scripts representing 7 basic emotions (happy, sad, surprised, angry, anxious, revolted, neutral) are presented; at the end of each vignette, participants are asked to choose the perceived emotion of a specific given actor, among multiple options listed in the answer form. The second section, “social inference (minimal)”, aims to investigate the understanding of conversational meanings that are determined by paralinguistic cues. Here, participants watch 15 videoclips of sincere or sarcastic or paradoxical conversational daily-life exchanges; for each clip, individuals are asked to answer 4 comprehension questions, respectively assessing the actors’ beliefs, meaning, intentions, and feelings understanding. The last section, “social inference (enriched)”, evaluates the ability to use contextual knowledge, like visual and verbal information, to derive meaning. 16 videoclips are shown, each one including a literally untrue comment. For each vignette, participants are asked to answer 4 comprehension questions, respectively assessing, as in the previous section, the actors’ beliefs, meaning, intentions, and feelings understanding.

c. To test the ability of managing emotions, i.e., “to be open to feelings, and to modulate them in oneself and others so as to promote personal understanding and growth of regulating emotions in oneself and in one’s relationships with others”^27^, we employed the Branch 4 (managing emotions) of the Mayer–Salovey–Caruso Emotional Intelligence Test (MSCEIT)^27^ (Italian version at ^28^). MSCEIT Branch 4 aims to evaluate the ability of managing emotions by asking participants to read short stories about an imaginary person going through an emotionally difficult situation and to select what course of action, among several choices, would be the most effective for that given person to cope with the difficult emotions of the story. Participants can rate every possible action ranging on a Likert scale ranging from «Very ineffective» to «Very effective». MSCEIT scoring was based on the consensus scoring methods outlined in the manual.

**4. Machine learning strategy**

We employed a double cycle, repeated nested cross-validation (CV) framework^29, 30^, to avoid information leakage between training and test, and to enforce an unbiased estimation of classification generalizability. We split the data first into training and test sets on an outer (CV2) cycle, and then we split the resulting training folds again into an inner (CV1), training and test data cycle ^29, 31, 32^.

Within CV1, all features underwent a pre-processing pipeline, consisting of the following steps:

1. We scaled each variable to a 0-1 range to remove between features differences effect from the training sample information. The scaling parameters were then applied to the inner and outer CV cycles.
2. Scaled data entered a k-Nearest Neighbor imputation step to fill the missing values in the data ^29, 33, 34^. For each missing value of a given subject, we identified a subset of cases in the training data that provided values for the given variable and that had values in all the other variables. This way, we selected a source subset of subjects who were sorted for their similarity with the target subject using the Euclidean distance. Then, we computed the median of the given variable in the 7 nearest neighbors of given subjects and use this median value to fill the missing one. This process was repeated until the imputation for all missing values was completed, filling the respectively computed nearest-neighbor medians. For the imputation, we used the original, non-imputed training matrix as a reference.
3. Features were further preprocessed by correcting for age and gender status effects. Specifically, we employed partial correlation to regress out the variance associated with age and gender from the feature scores within each inner and outer CV fold^35^.
4. To understand the discriminative utility of the input variables within each unimodal classifier^36^, each feature included underwent a wrapper-based forward feature selection procedure^37-39^. Specifically, the procedure selected the most parsimonious subset of features within the variable pool that optimized the algorithm average classification performance across the CV1 training and testing data, providing maximum prognostic performance with the smallest amount of predictors. The wrapper used a linear Support Vector Machine (SVM)^40^ to estimate the discriminative value of each variable, extracting the most discriminatory feature and reiterating the process over the remaining variable pool to select the 2nd best performing variable, which was added to the first one. This reiteration process was repeated until the identification of the optimal variable subspace. To avoid overfitting, we stopped the forward feature when 80% of the variables had been discarded from the feature pool, i.e., when the top 20% of the variables had been extracted by the wrapper. The ranking of the probability to be selected for each feature from the complete pool of variables entered both the cognitive and socio-cognitive classifiers is represented in Supplementary Figure - SF1A-SF1B for the HC1-BD cohort and SF2A-SF2B for the HC2-SCZ cohort respectively.

The trained model was then applied to CV2 by preprocessing the best discriminative variables using the learned scaling, the k-Nearest Neighbor imputation of missing values and age and gender variables regress-out, and determining each validation individual’s outcome class (i.e., HC vs. BD/SCZ) through a majority voting procedure across all ensemble models. In other words, in each variable evaluation step in CV1, the SVM algorithm modeled linear relationships between features and classification labels (i.e., HC1/HC2 vs. BD/SCZ). In the linear kernel space, the separability between HC1/HC2-like and most BD/SCZ-like individuals (i.e., the Support Vectors) was maximized within a hyperplane optimized by the SVM. The trained hyperplane allowed the algorithm to predict subjects' classification of the inner CV1 cycle by projecting its data into the learned kernel space: the estimation of their geometric distance to the decision boundary finally resulted in both an individual decision value and a predicted classification label per participant.

The procedure described above was reiterated for every combination of the SVM parameters related to misclassification cost (C parameters) and kernel width (γ parameters), within a grid defined by the ranges C = [0.125 - 16] and γ = [3.0518-5 - 8].

**5. Classification models discriminating between bipolar disorder and schizophrenia patients**

To investigate any potential residual cognitive and socio-cognitive difference between schizophrenia and bipolar disorder, we have built new models aimed at directly discriminating between the two clinical groups (i.e., without any control group). To this aim, we have generated individual and stacking-based signatures aimed at classifying BD and SCZ, employing the very same machine learning pipeline used for generating HC1 vs. BD and HC2 vs. SCZ models (see Supplementary Information, Section 4).

**5.1. Supplementary results and discussion**

Results revealed that cognitive and socio-cognitive classifiers discriminated between the two clinical groups at chance level (for detailed statistics, see ST3). Differently, the stacking-based model was able to classify between BD and SCZ patients above chance level, but still with a 15-20% lower cross-validated Balanced Accuracy - BAC (64.4%) when compared with performance from HC1 vs. BD (BAC: 80%) and HC2 vs. SCZ (BAC: 84.4%) stacking-based models. Furthermore, correlation analyses between decision scores extracted from the best performing classifier (i.e., the stacking-based) decisions and medication dose for BD and SCZ revealed significant association (Spearman’s rho = 0.18, p=.02), highlighting that in this cohort the algorithm performance might be affected by drug treatment.

Taken together, findings from BD vs. SCZ models support the existence of a small extent of residual cognitive and socio-cognitive differences between schizophrenia and bipolar disorder, which may be also affected by psychopharmacological treatment. However, given the BAC drop compared with HC1 vs. BD and HC2 vs. SCZ models, as well as the association with psychopharmacological treatment, it is possible that cognitive and socio-cognitive differences revealed by the direct comparison between BD and SCZ are negligible compared with the overlaps described in the main text (see Results and Discussion section in the manuscript).

REFERENCES

**1.** First MB, Gibbon M. The Structured Clinical Interview for DSM-IV Axis I Disorders (SCID-I) and the Structured Clinical Interview for DSM-IV Axis II Disorders (SCID-II). *Comprehensive handbook of psychological assessment, Vol. 2: Personality assessment.* Hoboken, NJ, US: John Wiley & Sons, Inc.; 2004:134-143.

**2.** Fusar-Poli P, Borgwardt S, Bechdolf A, et al. The psychosis high-risk state: a comprehensive state-of-the-art review. *JAMA Psychiatry* Jan 2013;70(1):107-120.

**3.** Schultze-Lutter F, Addington J, Ruhrmann S, Klosterkötter JJRGF. Schizophrenia proneness instrument, adult version (SPI-A). 2007.

**4.** Miller TJ, McGlashan TH, Rosen JL, et al. Prodromal assessment with the structured interview for prodromal syndromes and the scale of prodromal symptoms: predictive validity, interrater reliability, and training to reliability. *Schizophr Bull* 2003;29(4):703-715.

**5.** Antonucci LA, Raio A, Pergola G, et al. Machine learning-based ability to classify psychosis and early stages of disease through parenting and attachment-related variables is associated with social cognition. *BMC Psychol* Mar 23 2021;9(1):47.

**6.** Edition FJAPA. Diagnostic and statistical manual of mental disorders. 2013;21(21):591-643.

**7.** Conners CK. The computerized continuous performance test. *Psychopharmacology bulletin* 1985;21(4):891-892.

**8.** Reitan RM. The relation of the trail making test to organic brain damage. *Journal of consulting psychology* Oct 1955;19(5):393-394.

**9.** Corrigan JD, Hinkeldey NS. Relationships between parts A and B of the Trail Making Test. *Journal of clinical psychology* Jul 1987;43(4):402-409.

**10.** Grisetto F, Delevoye-Turrell YN, Roger C. Slower adaptation of control strategies in individuals with high impulsive tendencies. *Scientific reports* Oct 13 2021;11(1):20368.

**11.** Kreutzer JS, DeLuca J, Caplan B. *Encyclopedia of clinical neuropsychology*: Springer; 2011.

**12.** Caltagirone C, Gainotti G, Carlesimo GA, Parnetti L. Batteria per la valutazione del deterioramento mentale: I. Descrizione di uno strumento di diagnosi neuropsicologica. [The Mental Deterioration Battery: I. Description of a neuropsychological diagnostic instrument.]. *Archivio di Psicologia, Neurologia e Psichiatria* 1995;56(4):461-470.

**13.** Carlesimo GA, Caltagirone C, Gainotti G, Nocentini U. Batteria per la valutazione del deterioramento mentale: II. Standardizzazione e affidabilità diagnostica nell'identificazione di pazienti affetti da sindrome demenziale. [The Mental Deterioration Battery: II. Standardization and diagnostic reliability in the identification of demented patients.]. *Archivio di Psicologia, Neurologia e Psichiatria* 1995;56(4):471-488.

**14.** Bianchi A, Dai Pra M. Twenty years after Spinnler and Tognoni: new instruments in the Italian neuropsychologist's toolbox. *Neurol Sci* Sep 2008;29(4):209-217.

**15.** Uttl B, Graf P. The Wechsler memory scale-III: Validity and reliability. *Archives of Clinical Neuropsychology* 1999;14(8):705-706.

**16.** Babcock H, Levy L. *Test and manual of directions; the revised examination for the measurement of efficiency of mental functioning*. Wood Dale, IL, US: Stoelting; 1940.

**17.** Spinnler H, Tognoni G. Standardizzazione e taratura italiana di test neuropsicologici. *Masson Italia Periodici Press* 1987.

**18.** Rey A. L'examen clinique en psychologie. 1958.

**19.** Wechsler D. A Standardized Memory Scale for Clinical Use. *The Journal of Psychology* 1945/01/01 1945;19(1):87-95.

**20.** Kirchner WK. Age differences in short-term retention of rapidly changing information. *Journal of experimental psychology* Apr 1958;55(4):352-358.

**21.** Cowan N. What are the differences between long-term, short-term, and working memory? *Progress in brain research* 2008;169:323-338.

**22.** Grant DA, Berg E. A behavioral analysis of degree of reinforcement and ease of shifting to new responses in a Weigl-type card-sorting problem. *Journal of experimental psychology* 1948;38(4):404-411.

**23.** Kerr SL, Neale JM. Emotion perception in schizophrenia: specific deficit or further evidence of generalized poor performance? *J Abnorm Psychol* May 1993;102(2):312-318.

**24.** Rocca P, Galderisi S, Rossi A, et al. Social cognition in people with schizophrenia: a cluster-analytic approach. *Psychological medicine* Oct 2016;46(13):2717-2729.

**25.** Erol A, Putgul G, Kosger F, Ersoy B. Facial emotion recognition in schizophrenia: the impact of gender. *Psychiatry investigation* Mar 2013;10(1):69-74.

**26.** McDonald S, Bornhofen C, Shum D, Long E, Saunders C, Neulinger K. Reliability and validity of The Awareness of Social Inference Test (TASIT): a clinical test of social perception. *Disability and rehabilitation* Dec 30 2006;28(24):1529-1542.

**27.** Mayer J, Caruso D. Mayer-Salovey-Caruso Emotional Intelligence Test (MSCEIT). 2002.

**28.** D’Amico A, Curci AJF, Italy: OS. Traduzione ed adattamento italiano del Mayer-Salovey-Caruso Emotional Intelligence Test (MSCEIT). 2011.

**29.** Koutsouleris N, Kahn RS, Chekroud AM, et al. Multisite prediction of 4-week and 52-week treatment outcomes in patients with first-episode psychosis: a machine learning approach. *Lancet Psychiatry* Oct 2016;3(10):935-946.

**30.** Koutsouleris N, Kambeitz-Ilankovic L, Ruhrmann S, et al. Prediction Models of Functional Outcomes for Individuals in the Clinical High-Risk State for Psychosis or With Recent-Onset Depression: A Multimodal, Multisite Machine Learning Analysis. *JAMA Psychiatry* Nov 1 2018;75(11):1156-1172.

**31.** Filzmoser P, Liebmann B, Varmuza KJJoCAJotCS. Repeated double cross validation. 2009;23(4):160-171.

**32.** Varma S, Simon R. Bias in Error Estimation When Using Cross-Validation for Model Selection.” BMC Bioinformatics, 7(1), 91. *BMC bioinformatics* 02/01 2006;7:91.

**33.** Troyanskaya O, Cantor M, Sherlock G, Brown P, Hastie T, Tibshirani R, Botstein D, Altman RB. Missing value estimation methods for DNA microarrays. *Bioinformatics* Jun 2001;17(6):520-525.

**34.** Vapnik VN. An overview of statistical learning theory. *IEEE Trans Neural Netw* 1999;10(5):988-999.

**35.** Haas SS, Antonucci LA, Wenzel J, et al. A multivariate neuromonitoring approach to neuroplasticity-based computerized cognitive training in recent onset psychosis. *Neuropsychopharmacology* Mar 2021;46(4):828-835.

**36.** Antonucci LA, Pergola G, Pigoni A, et al. A Pattern of Cognitive Deficits Stratified for Genetic and Environmental Risk Reliably Classifies Patients With Schizophrenia From Healthy Control Subjects. *Biol Psychiatry* Apr 15 2020;87(8):697-707.

**37.** Antonucci LA, Penzel N, Pergola G, et al. Multivariate classification of schizophrenia and its familial risk based on load-dependent attentional control brain functional connectivity. *Neuropsychopharmacology* Mar 2020;45(4):613-621.

**38.** Dwyer DB, Falkai P, Koutsouleris N. Machine Learning Approaches for Clinical Psychology and Psychiatry. *Annu Rev Clin Psychol* May 7 2018;14:91-118.

**39.** Saeys Y, Inza I, Larranaga P. A review of feature selection techniques in bioinformatics. *Bioinformatics* Oct 1 2007;23(19):2507-2517.

**40.** Noble WS. What is a support vector machine? *Nature biotechnology* Dec 2006;24(12):1565-1567.

**SUPPLEMENTARY TABLES**

**Supplementary Table 1 – ST1.** Detailed description of the cognitive and socio-cognitive classifiers which entered the machine learning algorithm.

| **Classifier** | **Feature N**  **within classifier** | **Test** | **Target Domain/ Risk Variable** | **Indices calculated for features input** |
| --- | --- | --- | --- | --- |
| **Cognitive** | 52 | **AX Continuous Performance Test** | *Sustained and Selective Attention* | Proportion of total context false alarms  AD false alarms  AD false alarms reaction times  AY errors proportion  AY false alarms  AY false alarms reaction times  BD false alarms  BD false alarms reaction times  BX false alarms  BX false alarms reaction times  Correct responses  Correct responses proportion  Correct responses reaction times  Number of missed responses  Proportion of total false alarms  Response criterion index  Sensitivity index based on total context false alarms  Sensitivity index based on total false alarms  Signal to noise sensitivity  Standardized response criterion index  Target stimolous sensitivity  Transformed response criterion index  Transformed. sensitivity index based on total context false alarms  Transformed sensitivity index based on total false alarms |
|  |  |  |  |  |
|  |  |  |  |  |
|  |  |  |  |  |
|  |  |  |  |  |
|  |  |  |  |  |
|  |  |  |  |  |
|  |  |  |  |  |
|  |  |  |  |  |
|  |  | **N-Back Test** | *Working Memory* | 0-back  (Correct responses accuracy [%], reaction times and efficiency)  1-back  (Correct responses accuracy [%], reaction times and efficiency)  2-back  (Correct responses accuracy [%], reaction times and efficiency)  Between load  (Mean correct responses accuracy [%], mean reaction times and mean efficiency) |
|  |  | **Babcock Story Recall Test** | *Verbal episodic learning* | Delayed recall accuracy  Immediate recall accuracy  Total accuracy |
|  |  | **Rey Auditory Verbal Learning Test** | *Verbal incidental learning* | Corrected delayed recall score  Raw delayed recall score  Corrected immediate recall score  Raw immediate recall score |
|  |  | **Trail Making Test**  **A and B versions** | *Speed of Processing, Visual Attention,*  *Shifting* | Part A time to completion (seconds)  Part B time to completion (seconds)  Cognitive Flexibility Index  (Part B – Part A) (seconds) |
|  |  | **Verbal Fluency Test** | *Phonological and semantic fluency* | Phonological fluency total number of words  Semantic fluency total number of words |
|  |  | **Wechsler Memory Scale -III** | *Verbal and Non-Verbal Memory* | Memory Quotient |
|  |  | **Wisconsin Card Sorting Test** | *Shiting, Abstract Reasoning* | Number of completed categories  Number of perseverative errors  Perseverative errors (%) |
|  |  |  |  |  |
| **Socio-cognitive** | 37 | **Facial Emotion Identification Test** | *Emotion processing and identification* | Anger correct responses (%)  Anger reaction times  Average reaction times  Total correct responses (%)  Disgust correct responses (%)  Disgust reaction times  Fear correct responses (%)  Fear reaction times  Female expressions correct responses (%)  Female expressions reaction times  Happiness correct responses (%)  Happiness reaction times  Male expressions correct responses (%)  Male expressions reaction times  Neutral correct responses (%)  Neutral reaction times  Sadness correct responses (%)  Sadness reaction times  Surprise correct responses (%)  Surprise reaction times  Time to completion |
|  |  |  |  |  |
|  |  | **Mayer-Salovey-Caruso Emotional Intelligence test – Branch 4** | *Emotion management* | Emotion management (raw)  Emotion management (standardized)  Emotional reactions (raw)  Emotional reactions (standardized)  Global emotion management (raw)  Global emotion management (standardized) |
|  |  | **The Awareness of Social Inference Test** | *Social inference* | Negative emotions correct responses  Positive emotions correct responses  Positive+negative emotions correct responses  Sincere correct responses  Simple sarcasm correct responses  Complex sarcasm correct responses  Sincere + sarcasm correct responses  Paradoxical sarcasm correct responses  Lie correct responses |
|  |  |  |  | Lie + sarcasm correct responses |

**Supplementary Table 2A – ST2A.** Mean and standard deviation values for each cognitive feature in each study group.

| Cognitive features | HC1 (mean±SD) | BD (mean±SD) | HC2 (mean±SD) | SCZ (mean±SD) | CHR (mean±SD) | FEP (mean±SD) |
| --- | --- | --- | --- | --- | --- | --- |
| AX CPT_AY errors proportion | 0.03±0.05 | 0.17±0.26 | 0.05±0.15 | 0.13±0.22 | 0.11±0.19 | 0.25±0.38 |
| AX CPT_AD false alarms | 0.61±1.28 | 12.86±29.01 | 0.98±3.5 | 10.75±30.38 | 2.77±3.89 | 13.97±29.15 |
| AX CPT_AD false alarms reaction times | 20.04±188.17 | 6.22±12 | 0.88±2.08 | 3.41±7.51 | 42.79±201.05 | 3.22±11.19 |
| AX CPT_AY errors proportion | 0.08±0.08 | 0.14±0.18 | 0.09±0.17 | 0.16±0.19 | 0.16±0.16 | 0.19±0.21 |
| AX CPT_AY false alarms | 1.88±1.93 | 2.75±2.43 | 1.75±2.01 | 3.45±3.52 | 3.26±2.59 | 4.52±4.52 |
| AX CPT_AY false alarms reaction times | 1.19±1.91 | 1.64±2.24 | 0.94±1.76 | 1.63±2.43 | 5.98±22.44 | 7.31±29.84 |
| AX CPT_BD false alarms | 0.02±0.14 | 0.54±1.46 | 0.07±0.6 | 0.47±1.46 | 0.11±0.47 | 5.93±26.09 |
| AX CPT_BD false alarms reaction times | 0.01±0.05 | 0.63±1.73 | 0.03±0.25 | 0.27±1.23 | 0.37±1.59 | 14.68±74.02 |
| AX CPT_BX false alarms | 0.69±1.27 | 4.12±6.03 | 0.84±2.19 | 3.14±5.42 | 2.4±3.91 | 5.66±8.45 |
| AX CPT_BX false alarms reaction times | 0.73±1.54 | 2.01±2.62 | 0.75±1.88 | 1.83±2.68 | 1.84±2.67 | 1.87±2.41 |
| AX CPT_correct responses | 175.12±4.7 | 149.35±32.11 | 171.24±18.84 | 146.92±38.87 | 161.83±23.16 | 145.93±37.27 |
| AX CPT_correct responses proportion | 0.98±0.02 | 0.84±0.18 | 0.96±0.1 | 0.82±0.22 | 0.9±0.13 | 0.84±0.17 |
| AX CPT_correct responses reaction times | 55.74±14.81 | 52.61±14.48 | 54.32±15.29 | 54.16±17.2 | 48.49±19.36 | 54.18±16.92 |
| AX CPT_number of missed responses | 3.56±4.23 | 29.33±32.43 | 6.18±13.86 | 30.76±38.5 | 17.97±23.55 | 29.03±30.3 |
| AX CPT_proportion of total false alarms | 0.06±0.06 | 0.15±0.16 | 0.07±0.14 | 0.15±0.18 | 0.14±0.15 | 0.22±0.23 |
| AX CPT_response criterion index | 2.46±6 | 2.62±6.8 | 2.33±6.71 | 6.43±20.3 | 1.13±1.86 | 1.97±2.9 |
| AX CPT_sensitivity index based on proportion of total context false alarms | 0.99±0.02 | 0.89±0.19 | 0.98±0.08 | 0.89±0.15 | 0.93±0.1 | 0.91±0.23 |
| AX CPT_sensitivity index based on proportion of total false alarms | 0.98±0.02 | 0.9±0.11 | 0.97±0.05 | 0.9±0.1 | 0.93±0.09 | 0.87±0.12 |
| AX CPT_signal to noise sensitivity | 4.14±0.92 | 2.56±1.29 | 4.07±1.14 | 2.65±1.29 | 2.9±1.22 | 2.4±1.62 |
| AX CPT_standardized response criterion index | -0.21±0.42 | 0.05±0.48 | -0.14±0.43 | 0.09±0.66 | -0.15±0.36 | -0.08±0.56 |
| AX CPT_target stimolous sensitivity | 4.76±1.1 | 2.75±1.64 | 4.62±1.23 | 2.98±1.69 | 3.49±1.67 | 2.62±1.68 |
| AX CPT_transformed response criterion index | -0.77±1.74 | 0.12±1.02 | -0.58±1.56 | 0.22±1.54 | -0.47±1.02 | 0.02±1.1 |
| AX CPT_transformed sensitivity index based on proportion of total context false alarms | 2.96±0.14 | 2.5±0.55 | 2.92±0.26 | 2.58±0.46 | 2.71±0.35 | 2.44±0.62 |
| AX CPT_transformed sensitivity index based on proportion of total false alarms | 2.89±0.13 | 2.56±0.34 | 2.86±0.25 | 2.57±0.32 | 2.66±0.3 | 2.49±0.38 |
| BSRT_delayed recall accuracy | 7.18±1.14 | 5.73±2.16 | 7.03±1.6 | 5.4±2.07 | 6.67±1.42 | 4.32±2.8 |
| BSRT_immediate recall accuracy | 6.63±1.41 | 5.16±1.79 | 6.49±1.52 | 4.47±2.12 | 6±1.63 | 4.48±2.07 |
| BSRT_total accuracy | 13.81±2.17 | 10.89±3.66 | 13.53±2.55 | 9.88±3.79 | 12.67±2.78 | 8.8±4.5 |
| N-BACK_between load efficiency | 0.21±0.07 | 0.11±0.05 | 0.21±0.16 | 0.12±0.06 | 0.15±0.02 | 0.13±0.02 |
| N-BACK_between load mean accuracy | 93.69±5.81 | 72.82±17.73 | 92.06±7.62 | 75.18±15.71 | 79.5±3.11 | 79.18±4.2 |
| N-BACK_between load mean reaction times | 556.95±166.48 | 816.07±261.95 | 561.43±154.4 | 788.54±219.66 | 719.79±58.72 | 721.81±97.27 |
| N-BACK_one-back accuracy | 97.54±7.69 | 70.54±29.03 | 94.74±12.94 | 72.41±25.64 | 79.15±25.12 | 73.55±26.09 |
| N-BACK_one-back efficiency | 0.22±0.09 | 0.12±0.09 | 0.26±0.49 | 0.11±0.1 | 0.15±0.11 | 0.11±0.08 |
| N-BACK_one-back reaction times | 556.85±265.88 | 848.91±370.44 | 532.62±268.9 | 856.29±316.79 | 768.43±354.13 | 890.59±365.04 |
| N-BACK_two-back accuracy | 83.76±19.05 | 53.04±20.23 | 81.98±20.7 | 53.71±24.23 | 63.24±26.56 | 54.38±21.71 |
| N-BACK_two-back efficiency | 0.19±0.13 | 0.08±0.06 | 0.19±0.12 | 0.08±0.07 | 0.14±0.13 | 0.08±0.07 |
| N-BACK_two-back reaction times | 602.05±296.26 | 860.41±317.7 | 596.22±289.8 | 830.97±287.03 | 700.71±389.23 | 874.97±371.92 |
| N-BACK_zero back reaction times | 523.2±122.36 | 759.93±212.89 | 545.59±134.1 | 690.69±195.47 | 614.54±189.94 | 692.45±211.77 |
| N-BACK_zero-back accuracy | 100±0 | 96.06±13.86 | 99.96±0.51 | 99.13±5.22 | 99.71±1.29 | 97.78±9.37 |
| N-BACK_zero-back efficiency | 0.2±0.05 | 0.13±0.04 | 0.19±0.05 | 0.16±0.05 | 0.18±0.05 | 0.15±0.05 |
| PHONOLOGICAL FLUENCY_total number of words | 14.48±3.27 | 10.17±3.55 | 14.78±4.25 | 9.6±2.93 | 10.49±4.21 | 9.2±3.28 |
| RAVLT_delayed recall number of words (corrected) | 9.47±2.12 | 7.05±3.03 | 10.2±2.05 | 6.25±3.47 | 7.91±2.97 | 32.6±127.9 |
| RAVLT_delayed recall number of words (raw) | 12.92±2.05 | 8.98±3.27 | 13.31±1.93 | 8.52±3.53 | 11.24±3.11 | 8.73±3.37 |
| RAVLT_immediate recall number of words (corrected) | 47.85±6.94 | 35.43±9.61 | 47.54±8.36 | 32.38±10.81 | 40.38±9.86 | 32.27±12.78 |
| RAVLT_immediate recall number of words (raw) | 58.25±7.42 | 41.45±10.67 | 57.71±9.28 | 40.38±11.31 | 50.52±10.48 | 41.73±12.97 |
| SEMANTIC FLUENCY_total number of words | 25.29±4.83 | 17.97±5 | 23.75±4.83 | 15.74±4.77 | 17.46±4.52 | 16.01±4.6 |
| TMT A_time to completion | 24±8.1 | 38.92±22.09 | 23.13±7.64 | 42.15±17.09 | 36±19.35 | 40.03±18.82 |
| TMT B_time to completion | 51.97±21.26 | 101.93±63.92 | 56.15±35.55 | 106.2±50.41 | 96.38±59.75 | 96.97±52.79 |
| TMT B-A_time to completion | 27.97±18.64 | 63.52±47.63 | 33.13±33.4 | 64.34±44.71 | 61.06±48.85 | 56.93±42.37 |
| WCST_number of completed categories | 5.63±1.18 | 4.09±2 | 5.6±1.14 | 3.47±2.26 | 5.03±1.52 | 4.66±2.14 |
| WCST_perseverative errors (%) | 9.8±6.82 | 20.49±14.69 | 11.56±10.82 | 21.53±14.41 | 21.46±19.13 | 16.04±9.04 |
| WCST_perseverative errors | 9.43±9.43 | 23.23±13.81 | 10.45±8.93 | 26.07±19.55 | 23.26±21.16 | 17.14±11.73 |
| WMS_memory quotient | 104.62±12.27 | 86.28±17.26 | 104.39±12.41 | 84.18±15.24 | 82.64±18.17 | 83.26±15.78 |

**Abbreviations:** BD=patients with Bipolar Disorder; BSRT=Babcock Story Recall Test; CHR=individuals at Clinical High Risk; corr.=corrected; CPT=Continuous Performance Test; FEP=individuals at First Episode of Psychosis; HC1=Healthy Controls (group 1); HC2=Healthy Controls (group 2); RAVLT=Rey Auditory Verbal Learning Test; SCZ=patients with Schizophrenia; TMT A/B/B-A=Trail Making Test – Part A/Part B/Part B-Part A; WCST=Wisconsin Card Sorting Test; WMS=Wechsler Memory Scale.

**Supplementary Table 2B – ST2B.** Mean and standard deviation (SD) values for each socio-cognitive feature in each study group.

| Socio-cognitive feature | HC1 (mean±SD) | BD (mean±SD) | HC2 (mean±SD) | SCZ (mean±SD) | CHR (mean±SD) | FEP (mean±SD) |
| --- | --- | --- | --- | --- | --- | --- |
| FEIT_anger correct responses (%) | 85.25 ± 14.49 | 65.97 ± 21.17 | 82.39 ± 16.26 | 65.03 ± 23.53 | 77.78 ± 13.5 | 57.7 ± 27.95 |
| FEIT_anger response time | 3679.85 ± 1836.5 | 5080.11 ± 1861.78 | 4054.62 ± 1963.74 | 4968.67 ± 2305.63 | 5155.74 ± 2011.62 | 5156.84 ± 2223.29 |
| FEIT_average response time | 3476.02 ± 1105.13 | 4561.62 ± 1633.83 | 3744.31 ± 1377.26 | 7848.01 ± 34050.88 | 4728.66 ± 1594.08 | 4436.46 ± 1297.81 |
| FEIT_ correct responses (%) | 82.04 ± 10.15 | 71.87 ± 10.77 | 81.26 ± 8.28 | 68.95 ± 12.6 | 76.41 ± 7.12 | 69.76 ± 13.83 |
| FEIT_disgust correct responses (%) | 81.11 ± 16.95 | 75.84 ± 18.06 | 82.75 ± 16.08 | 70.08 ± 21.82 | 75.9 ± 17.6 | 70.32 ± 25.20 |
| FEIT_disgust response time | 3123.93 ± 2574.42 | 4312.32 ± 1861.7 | 3381.33 ± 1504.87 | 4354.74 ± 2158.84 | 4654.29 ± 2157.33 | 4514.47 ± 2119.95 |
| FEIT_fear correct responses (%) | 73.82 ± 21.13 | 56.91 ± 24.42 | 69.76 ± 21.31 | 52 ± 26.46 | 65 ± 22.85 | 52.88 ± 28.13 |
| FEIT_fear response time | 4262.8 ± 2706.94 | 5191.06 ± 2324.76 | 4463.63 ± 1747.1 | 5421.47 ± 2281.64 | 5164.83 ± 1539.33 | 4731.11 ± 1857.44 |
| FEIT_female expressions correct responses (%) | 84.38 ± 10.48 | 78.85 ± 10.13 | 84.9 ± 8.73 | 73.26 ± 13.26 | 80.57 ± 7.16 | 73.34 ± 14.17 |
| FEIT_female expressions response time | 3470.42 ± 1296.39 | 4465.81 ± 1553.65 | 3578.44 ± 1361.08 | 4570.82 ± 2074.99 | 4582.09 ± 1484.52 | 4557.6 ± 1513.99 |
| FEIT_happiness correct responses (%) | 86.41 ± 17.82 | 81.8 ± 19.02 | 87.66 ± 13.36 | 82.38 ± 17.83 | 86.99 ± 9.9 | 80.93 ± 20.14 |
| FEIT_happiness response time | 3120.26 ± 1244.05 | 3940.53 ± 1439.56 | 3256.16 ± 1302.97 | 3870.35 ± 1679.89 | 4018.23 ± 1507.45 | 3590.35 ± 1191.23 |
| FEIT_male expressions correct responses (%) | 78.91 ± 11.48 | 403.96 ± 1342.79 | 76.99 ± 10.77 | 63.95 ± 15.46 | 70.74 ± 10.79 | 61.47 ± 16.84 |
| FEIT_male expressions response time | 3488.23 ± 1106.23 | 4673.66 ± 1811.33 | 3973.58 ± 1496.1 | 4872.1 ± 2217.99 | 5071.43 ± 1785.27 | 4815.05 ± 1576.93 |
| FEIT_neutral correct responses (%) | 90.09 ± 14.24 | 86.92 ± 19.04 | 92.13 ± 13.66 | 81.89 ± 21.09 | 88.97 ± 11.02 | 90.1 ± 14.99 |
| FEIT_neutral response time | 3034.71 ± 1357.05 | 3954.32 ± 1992.46 | 3116.8 ± 1498.48 | 4442.14 ± 3402.68 | 4127.49 ± 1706.89 | 3801.89 ± 1393.47 |
| FEIT_sadness correct responses (%) | 64.42 ± 25.09 | 53.19 ± 21.57 | 59.92 ± 22.63 | 49 ± 25.25 | 50.71 ± 18.92 | 56.02 ± 23.61 |
| FEIT_sadness response time | 3805.97 ± 1304.82 | 5029.6 ± 2360.99 | 4481.85 ± 1836.65 | 5336.14 ± 2817.22 | 5626.2 ± 2348.18 | 4897.26 ± 2012.01 |
| FEIT_surprise correct responses (%) | 94.02 ± 13.24 | 82.66 ± 23.06 | 94.86 ± 13.06 | 81.1 ± 25.79 | 88.97 ± 17.36 | 83.5 ± 22.75 |
| FEIT_surprise response time | 3328.76 ± 2128.16 | 4223.45 ± 1803.25 | 3409.33 ± 1539.23 | 4522.19 ± 2898.08 | 4504.8 ± 2247.56 | 4237.42 ± 1881.03 |
| FEIT_time to completion | 202853.79 ± 182060.84 | 250915.72 ± 89863.06 | 206767.3 ± 75863.89 | 257645.63 ± 114683.42 | 260008.77 ± 87505.58 | 244156.54 ± 71501.76 |
| MSCEIT_emotion management (raw) | 0.37 ± 0.06 | 0.32 ± 0.07 | 0.35 ± 0.07 | 0.31 ± 0.08 | 0.32 ± 0.07 | 0.47 ± 0.60 |
| MSCEIT_emotion management (standardized) | 88.37 ± 8.64 | 79.18 ± 9.5 | 83.9 ± 12.46 | 69.17 ± 8.62 | 86.18 ± 11.26 | 86.85 ± 8.59 |
| MSCEIT_emotional reactions (raw) | 0.37 ± 0.09 | 0.3 ± 0.1 | 0.37 ± 0.09 | 0.28 ± 0.1 | 0.34 ± 0.08 | 0.4 ± 0.47 |
| MSCEIT_emotional reactions (standardized) | 88.99 ± 9 | 80.95 ± 12.56 | 88.11 ± 10.87 | 78.3 ± 12.78 | 91.72 ± 11.12 | 94.75 ± 11.89 |
| MSCEIT_global emotion management (raw) | 0.37 ± 0.07 | 0.31 ± 0.08 | 0.36 ± 0.07 | 0.3 ± 0.09 | 0.33 ± 0.07 | 0.38 ± 0.45 |
| MSCEIT_global emotion management (standardized) | 87.12 ± 9.23 | 77.13 ± 11.39 | 84.30 ± 11.97 | 78.41 ± 8.45 | 82.66 ± 15.80 | 72.77 ± 18.20 |
| TASIT I_negative emotions correct responses | 15.15 ± 1.21 | 12.65 ± 2.93 | 14.91 ± 1.17 | 12.97 ± 2.53 | 14.21 ± 1.74 | 13.9 ± 2.09 |
| TASIT I_positive + negative emotions correct responses | 25.89 ± 1.6 | 21.71 ± 4.68 | 25.38 ± 2.17 | 22.83 ± 3.8 | 24.32 ± 2.24 | 24 ± 3.32 |
| TASIT I_positive emotion correct responses | 10.79 ± 1.05 | 9.06 ± 2.18 | 10.55 ± 1.4 | 9.84 ± 1.8 | 10.12 ± 1.41 | 10.29 ± 1.61 |
| TASIT II_paradoxical sarcasm correct responses | 18.11 ± 2.1 | 12.42 ± 4.51 | 17.74 ± 3.83 | 11.42 ± 5.34 | 14.62 ± 3.69 | 12 ± 3.834 |
| TASIT II_simple sarcasm correct responses | 18.21 ± 2.06 | 14.33 ± 4.78 | 17.78 ± 2.71 | 12.9 ± 4.81 | 16.56 ± 2.5 | 13.67 ± 2.80 |
| TASIT II_sincere + correct responses | 53.18 ± 4.58 | 42.53 ± 10.89 | 51.74 ± 5.99 | 39.62 ± 9.14 | 48.29 ± 4.91 | 42.33 ± 7.02 |
| TASIT II_sincere correct responses | 16.97 ± 2.74 | 16.61 ± 2.24 | 16.78 ± 2.76 | 16.25 ± 2.94 | 16.71 ± 2.3 | 16.43 ± 2.82 |
| TASIT III_lie + sarcasm correct responses | 52.55 ± 4.54 | 43.04 ± 9.22 | 51.85 ± 4.8 | 40.24 ± 8.01 | 46.38 ± 7.23 | 41.05 ± 6.29 |
| TASIT III_lie correct responses | 25.01 ± 3.25 | 21.9 ± 4.59 | 24.96 ± 3.52 | 20.07 ± 4.39 | 22.56 ± 3.61 | 20.95 ± 3.53 |
| TASIT III_sarcasm correct responses | 27.53 ± 2.83 | 21.15 ± 5.9 | 27.08 ± 3.22 | 19.82 ± 5.41 | 23.53 ± 4.86 | 20.1 ± 4.46 |

**Abbreviations**: BD=patientis with Bipolar Disorder; CHR=individuals at Clinical High Risk; FEIT=Facial Emotion Identification Test; FEP=individuals at First Episode of Psychosis; HC1=Healthy Controls (group 1); HC2=Healthy Controls (group 2); MSCEIT=Mayer-Salovey-Caruso Emotional Intelligence Test; SCZ=patients with Schizophrenia; std.=standardized; TASIT=The Awareness of Social Inference Test.

**Supplementary Table 3 – ST3**: Validated classification performance of unimodal and stacking-based classifiers in the Bipolar Disorder – Schizophrenia patients cohort.

| Classification: BD-SCZ cohort | | | | | | | | | | | | |
| --- | --- | --- | --- | --- | --- | --- | --- | --- | --- | --- | --- | --- |
|  | **True Negatives** | **True Positives** | **False Negatives** | **False Positives** | **Sensitivity** | **Specificity** | **Balanced Accuracy** | **Area Under the Curve** | **Positive Predictive Value** | **Negative Predictive Value** | **Positive Likelihood**  **Ratio** |  |
| Cognitive classifier | 34 | 69 | 49 | 25 | 58.5 | 57.6 | 58.1 | 0.62 | 73.4 | 41 | 1.4 |  |
| Socio-cognitive classifier | 35 | 70 | 48 | 24 | 59.3 | 59.3 | 59.3 | 0.63 | 74.5 | 42.2 | 1.5 |  |
| Stacking-based  classifier | 36 | 80 | 38 | 23 | 67.8 | 61 | 64.4 | 0.67 | 77.7 | 48.6 | 1.7 |  |

**Abbreviations**: BD=patients with Bipolar Disorder; SCZ=patients with Schizophrenia.

**SUPPLEMENTARY FIGURES**

**Supplementary Figure 1A - SF1A.** Probability of each feature for being selected in the Machine Learning Cross-Validation framework for the cognitive classifier discriminating Healthy Controls (group 1) vs. Bipolar Disorder patients. Score closer to 1 represent a higher probability of being selected for decision by the Support Vector Machine algorithm.


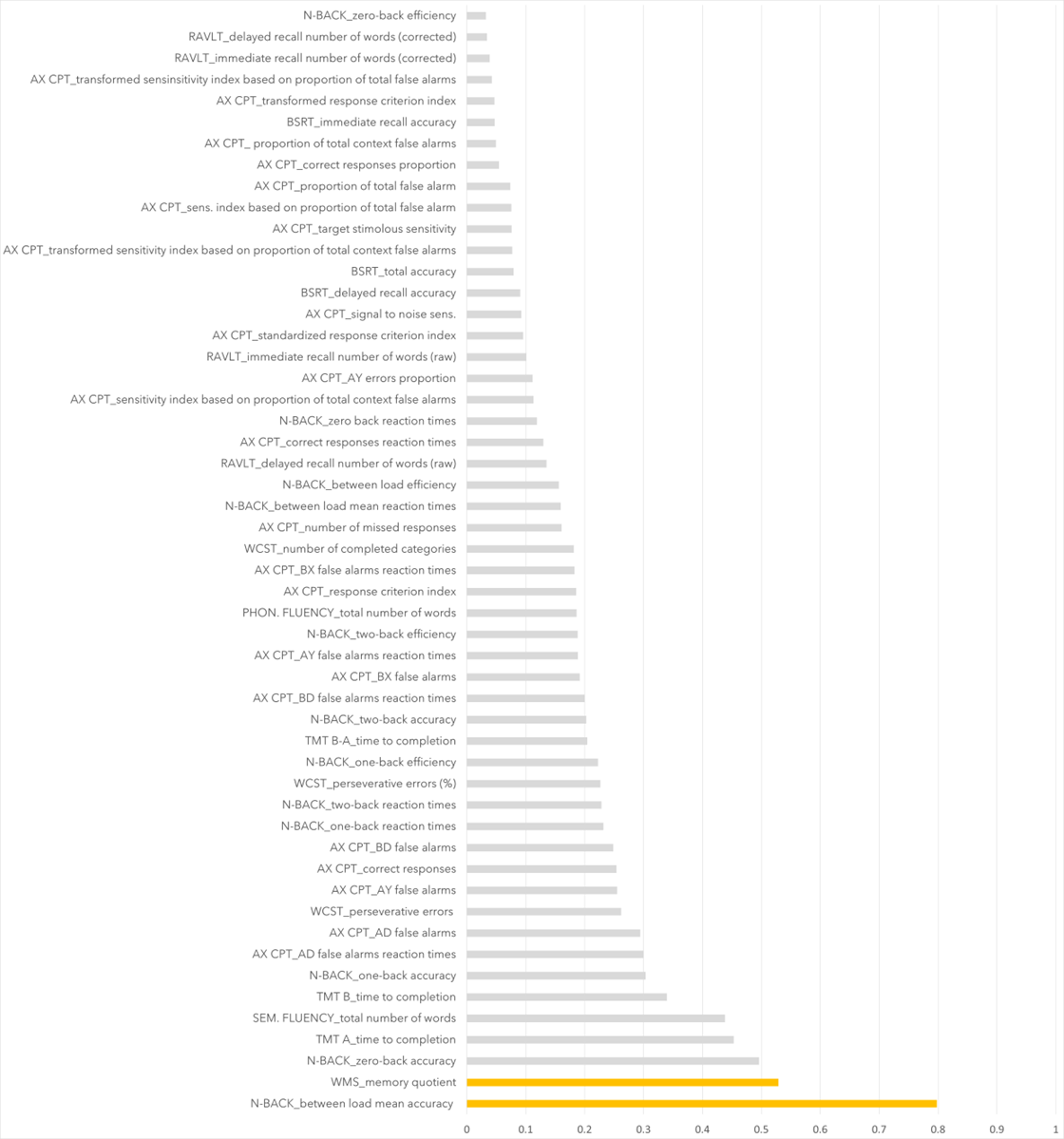


**Abbreviations:** BSRT=Babcock Story Recall Test; CPT=Continuous Performance Test; N=Number; RAVLT=Rey Auditory Verbal Learning Test; TMT A/B/B-A=Trail Making Test – Part A/Part B/Part B-Part A; WCST=Wisconsin Card Sorting Test; WMS=Wechsler Memory Scale.


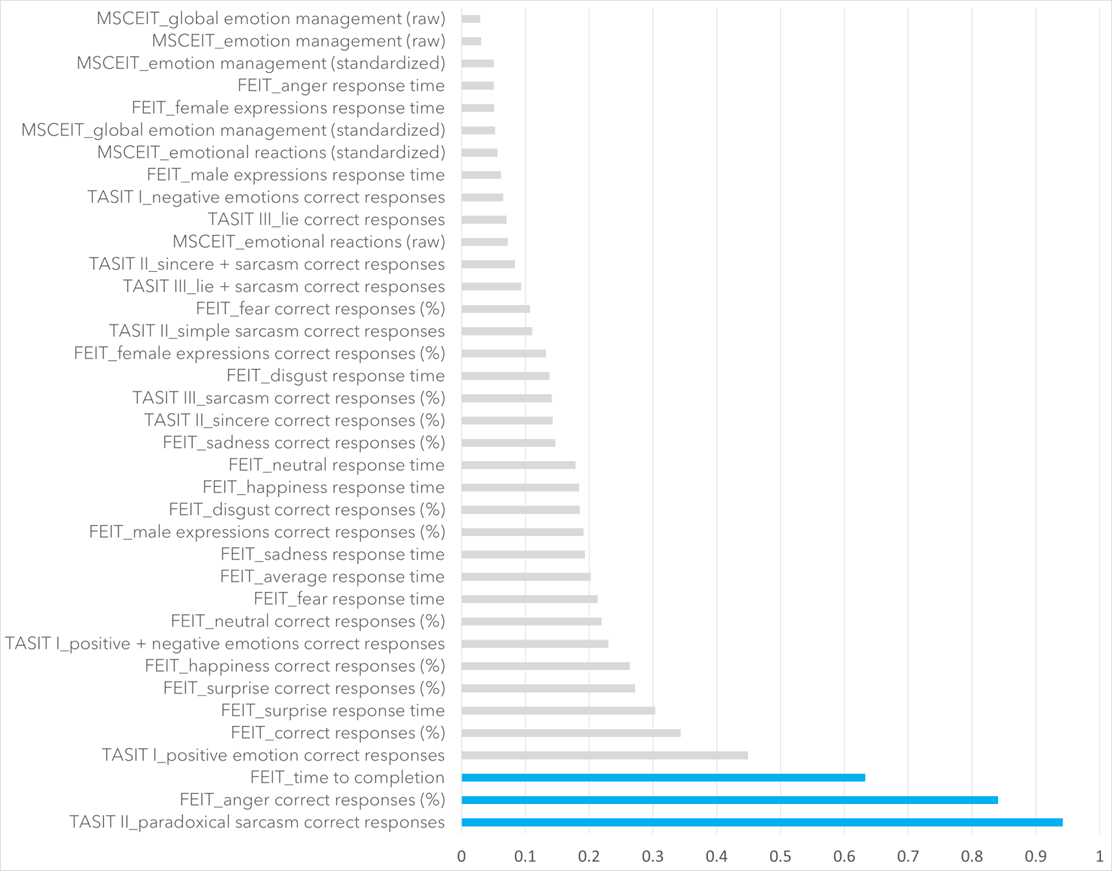
**Supplementary Figure 1B - SF1B.** Probability of each feature for being selected in the Machine Learning Cross-Validation framework for the socio-cognitive classifier discriminating Healthy Controls (group 1) vs. Bipolar Disorder patients. Score closer to 1 represent a higher probability of being selected for decision by the Support Vector Machine algorithm.

**Abbreviations:** FEIT=Facial Emotion Identification Test; MSCEIT=Mayer-Salovey-Caruso Emotional Intelligence Test; TASIT=The Awareness of Social Inference Test.

**Supplementary Figure 2A - SF2A.** Probability of each feature for being selected in the Machine Learning Cross-Validation framework for the cognitive classifier discriminating Healthy Controls (group 2) vs. Schizophrenia patients. Score closer to 1 represent a higher probability of being selected for decision by the Support Vector Machine algorithm.


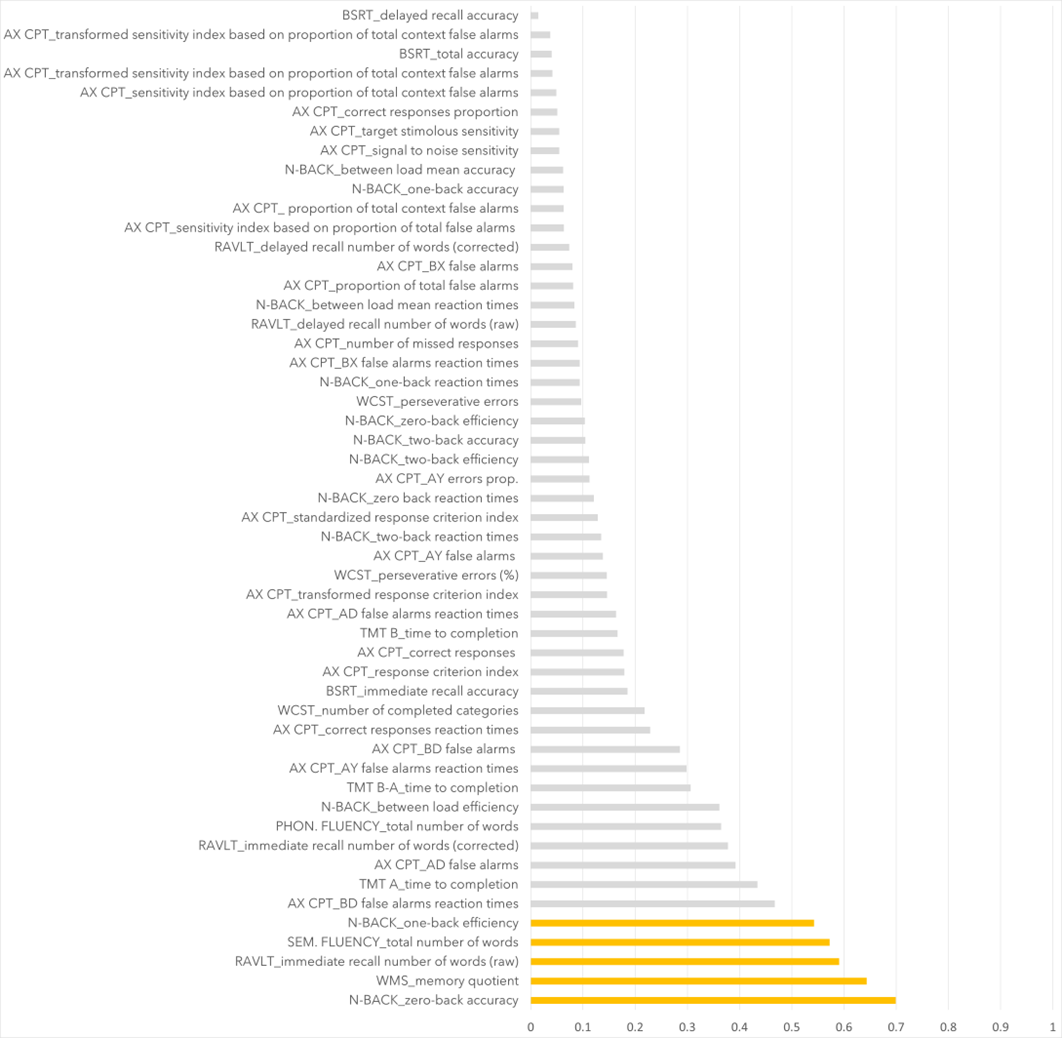


**Abbreviations:** BSRT=Babcock Story Recall Test; CPT=Continuous Performance Test; N=Number; RAVLT=Rey Auditory Verbal Learning Test; TMT A/B/B-A=Trail Making Test – Part A/Part B/Part B-Part A; WCST=Wisconsin Card Sorting Test; WMS=Wechsler Memory Scale.

**Supplementary Figure 2B – SF2B.** Probability of each feature for being selected in the Machine Learning Cross-Validation framework for the socio-cognitive classifier discriminating Healthy Controls (group 2) vs. Schizophrenia patients. Score closer to 1 represent a higher probability of being selected for decision by the Support Vector Machine algorithm.


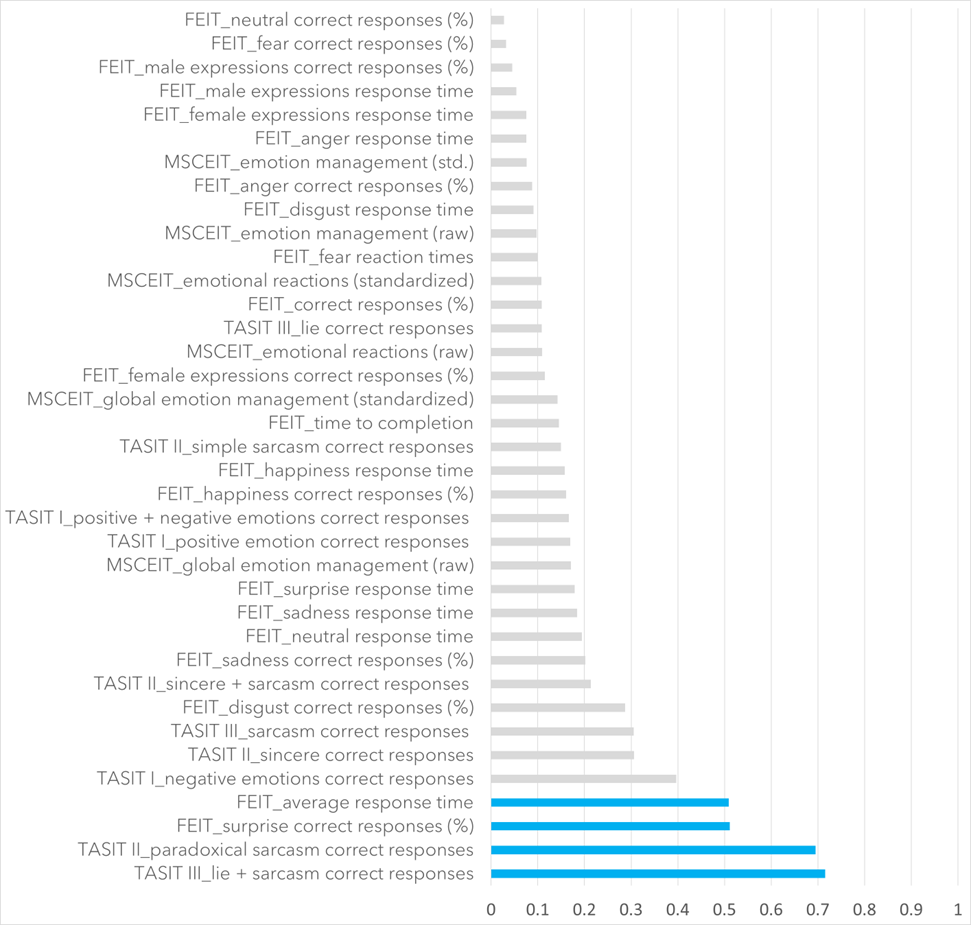


**Abbreviations:** FEIT=Facial Emotion Identification Test; MSCEIT=Mayer-Salovey-Caruso Emotional Intelligence Test; TASIT=The Awareness of Social Inference Test.
